# An optimization-based waypoint generation method for overcoming local minima in artificial potential field navigation

**DOI:** 10.1371/journal.pone.0360512

**Published:** 2026-10-21

**Authors:** Avinesh Prasad, Vishal Chand, Sushita Sharma, Priynka Sharma, Sandeep Ameet Kumar

**Affiliations:** The University of the South Pacific, Suva, Fiji Islands; Beijing Institute of Technology, CHINA

## Abstract

The Artificial Potential Field (APF) method is widely used in robot path planning due to its simplicity and computational efficiency. However, it is well known to suffer from the local minima problem, where the resultant force acting on the robot becomes zero at non-goal configurations, leading to motion stagnation in cluttered environments. This paper proposes an optimization-based waypoint generation framework to address the local minima issue in APF navigation while ensuring explicit collision avoidance. When the direct path between the robot and the target is infeasible due to obstacle interference, intermediate waypoints are generated by solving a constrained optimization problem that guides the robot along a locally defined reference direction while enforcing obstacle clearance constraints for each path segment. Unlike conventional APF modifications that rely on artificial perturbation forces or random escape strategies, the proposed method constructs collision-free intermediate waypoints through finite candidate evaluation and explicit segment-wise collision checking, enabling the robot to avoid obstacles and overcome local-minima while preserving goal-directed motion and ensuring convergence to the target. The effectiveness of the proposed approach is validated through numerical simulations on two robotic platforms, namely a point-mass robot and a wheelchair-type robotic model. The results demonstrate that the method successfully resolves local minima situations, ensures collision-free motion, and consistently guides the robots to their target locations in complex environments. Furthermore, the framework is general and can be applied to different robotic systems.

## Introduction

Robots play a vital role in a wide range of industries and applications. The use of robots continues to expand as technology advances, offering new possibilities in transportation, manufacturing, agriculture, healthcare, surveillance, exploration, construction, and domestic environments. Robots are particularly useful in performing dull, dirty, and dangerous tasks that may pose significant risks to human operators. Mobile robots, including autonomous ground vehicles, unmanned aerial vehicles (UAVs), planetary rovers, underwater robots, and service robots, have attracted considerable research interest due to their ability to operate autonomously in dynamic and uncertain environments [1,2]. Their ability to perceive, interpret, and interact with their surroundings enables them to perform a variety of navigation and decision-making tasks. Mobile robots are increasingly being deployed in automated warehouses, elderly care facilities, industrial production lines, hazardous environments, and planetary exploration missions [3,4].

Recent studies have investigated continuous robot operation using dynamic visual servo control, highlighting the importance of continuous feedback and adaptive control in changing environments [5]. Such approaches are particularly relevant to agricultural applications, including fruit harvesting, where robots must operate continuously while responding to variations in the workspace. More recently, collision-free path planning for agricultural robots has also been explored using recurrent deep reinforcement learning, demonstrating the potential of learning-based approaches for generating safe navigation behaviours in complex harvesting environments [6]. Beyond agricultural robotics, recent advances in path planning have incorporated diverse predictive, data-driven, and generative approaches. These include statistics-based and learning-based methods for multi-objective shortest-path cost prediction, receding-horizon strategies for collision avoidance, and visual generative adversarial networks for end-to-end trajectory generation in autonomous systems [7–9]. Collectively, these developments demonstrate the increasing research interest in reliable, adaptive, and collision-free path planning and robot operation in dynamic and unstructured environments.

Robot navigation remains one of the most fundamental problems in mobile robotics. The objective of navigation is to determine a feasible and efficient path from an initial position to a target destination while avoiding obstacles in the environment [4,10]. Over the past several decades, numerous navigation methods have been developed and can generally be classified as classical, heuristic, optimization-based, and hybrid approaches. Each category offers unique advantages and limitations in terms of computational complexity, optimality, robustness, and real-time applicability.

Optimization-based techniques have received significant attention for robot navigation and path planning problems. Jalali et al. [11] employed four bio-inspired evolutionary algorithms - Multi-Verse Optimizer (MVO), Moth-Flame Optimization (MFO), Cuckoo Search (CS), and Particle Swarm Optimization (PSO) – to optimize multilayer perceptron (MLP) neural networks for robot navigation, incorporating evolutionary population dynamics and mutation operators to balance exploration and exploitation during optimization. Liu et al. [12] improved mobile robot path planning by using Delaunay triangulation to process complex obstacles and generate Voronoi points as prioritized nodes for pathfinding, while also extracting obstacle edges via grid representations; an enhanced A* search algorithm was then applied to compute both priority and regular paths for effective obstacle avoidance. Oliveira et al. [13] utilized Bayesian Optimization together with Gaussian Process models to enable safe robot navigation in uncertain outdoor environments by learning terrain traversability and supporting informed decision-making during exploration. More recently, metaheuristic and learning-based optimization techniques have demonstrated promising results in complex navigation scenarios [14,15].

Waypoint generation has emerged as an effective strategy for guiding robots through complex environments. Waypoints act as intermediate navigation targets that assist robots in avoiding obstacles while maintaining progress toward the final destination [16–20]. Moreno et al. [16] proposed an automatic waypoint generation approach to facilitate navigation through narrow passages by identifying difficult regions and generating auxiliary navigation targets. Eslamiat et al. [21] developed a deep reinforcement learning framework for autonomous waypoint planning and trajectory generation for UAVs. Although waypoint-based approaches have shown considerable promise, most existing studies focus primarily on obstacle avoidance and path feasibility rather than addressing the local minima problem associated with potential field methods.

Among the various classical navigation approaches identified in recent surveys [22], the Artificial Potential Field (APF) method proposed by Khatib [23] remains one of the most widely used techniques due to its conceptual simplicity, computational efficiency, and suitability for real-time applications. In the APF framework, attractive forces draw the robot toward the goal, while repulsive forces push it away from obstacles. Numerous extensions and improvements of the APF method have been proposed over the years to enhance navigation performance and obstacle avoidance capabilities [24,25].

Despite its advantages, the APF method suffers from several well-known limitations, including oscillatory behaviour near obstacles, difficulties in narrow passages, and the local minima problem [24,26]. Local minima occur when the resultant potential gradient becomes zero at a location other than the goal position, causing the robot to become trapped and preventing further progress toward the target. Such situations commonly arise when the robot, obstacle, and goal positions become collinear or when the environment contains closely spaced obstacles and narrow corridors. Fig 1 illustrates two representative local minima situations encountered by a point-mass robot navigating from the initial position (5,5) to the goal position (25,25).

**Fig 1:**
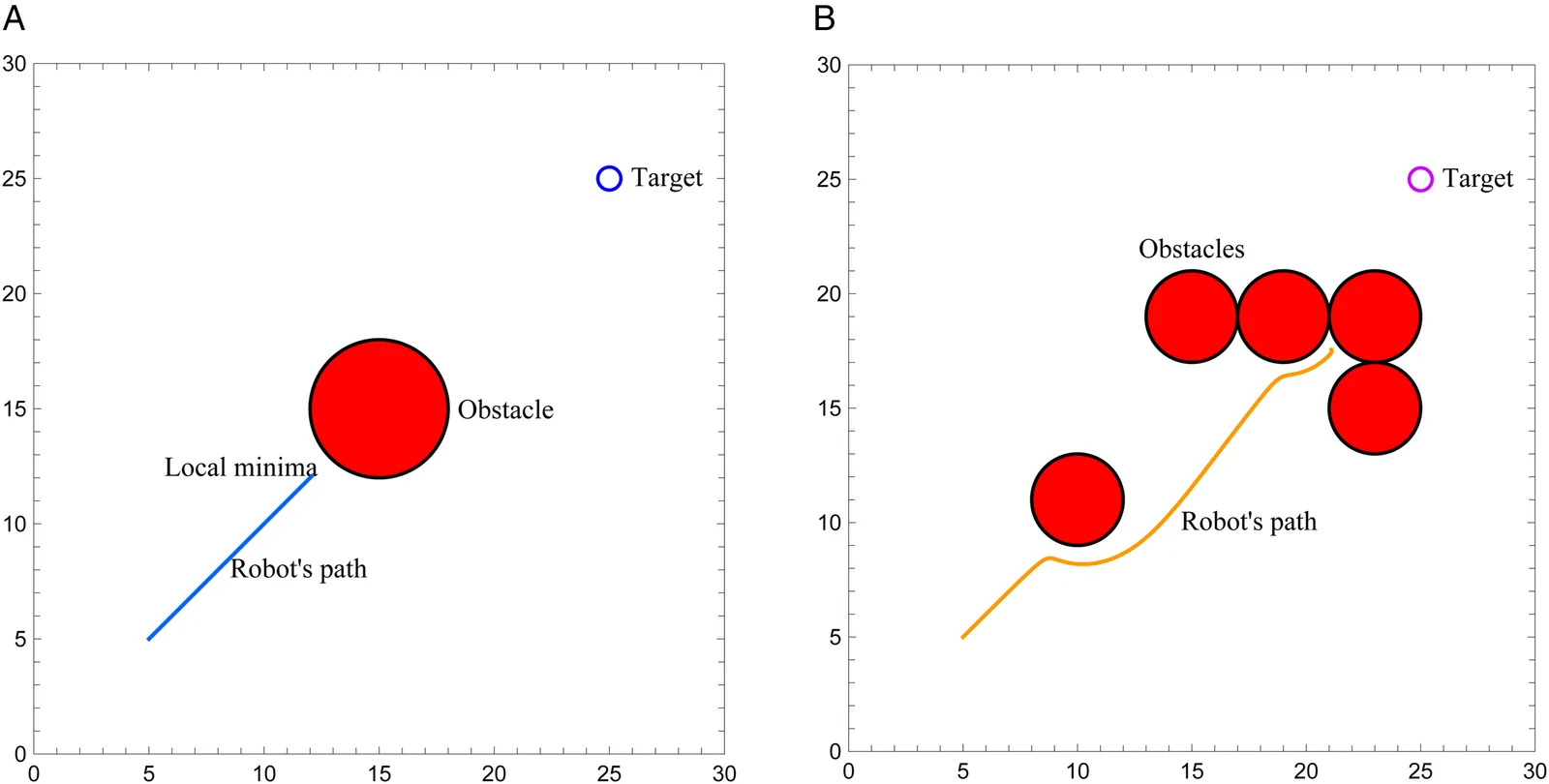
Local minima situations in the artificial potential field method.

Several approaches have been proposed to mitigate the local minima problem in artificial potential field (APF) navigation, including harmonic potential fields, virtual target points, wall-following strategies, fuzzy logic methods, evolutionary optimization techniques, learning-based mechanisms, and hybrid navigation frameworks [15,24,27,28]. However, most existing solutions either modify the underlying potential function or integrate APF with computationally intensive global planners, and they often require extensive parameter tuning, environment-specific adaptations, or increased computational effort. Despite these advances, relatively little attention has been given to optimization-generated waypoints as a systematic mechanism for overcoming APF local minima while preserving the simplicity and computational efficiency of the original APF framework.

The method proposed in this paper differs from existing local-minima avoidance techniques in three important aspects. First, instead of modifying the attractive and repulsive potential functions, the proposed framework preserves the original APF navigation structure and introduces a waypoint-generation mechanism that operates independently of the navigation controller. Second, the waypoint generation process is formulated as a constrained optimization problem that simultaneously considers progress toward the goal and obstacle-clearance requirements. Third, the generated waypoints are applicable to different robotic platforms without requiring modifications to the underlying control laws. Consequently, the proposed framework provides a computationally simple and structured mechanism for overcoming local minima while maintaining the attractive features of conventional APF navigation.

To the best of the authors’ knowledge, relatively little attention has been devoted to optimization-driven waypoint generation as a systematic mechanism for overcoming APF local minima while retaining the simplicity of the original APF formulation. This motivates the development of the proposed hybrid navigation framework.

To address this research gap, this paper proposes an optimization-based waypoint generation technique for robot navigation in obstacle-cluttered environments. The proposed method automatically generates intermediate waypoints that satisfy distance, directional, and collision-avoidance constraints. These waypoints serve as temporary navigation goals that guide the robot safely around obstacles and enable it to escape local minima regions. The main contributions of this paper are summarized as follows:

- An optimization-based waypoint generation algorithm is developed to automatically determine collision-free intermediate waypoints for mobile robot navigation.
- A hybrid navigation framework combining the proposed waypoint generation strategy with the Lyapunov-based Control Scheme (LbCS) [29] is developed to overcome local minima while maintaining safe navigation toward the target.
- The effectiveness of the proposed approach is demonstrated through simulations involving both point-mass and wheelchair robots, illustrating its applicability to robotic systems with different kinematic characteristics.

## Robot model and problem formulation

Consider a planar point-mass robot, represented as a disk of radius $r_{P}\geq 0$ with center position $𝐱(t)=(x(t),y(t))\in {\mathbb{R}}^{2}$ at time t≥0. The robot is defined as the set


$$\begin{array}{c} P(t)=\{𝐳\in {\mathbb{R}}^{2}:\|𝐳-𝐱(t|\leq r_{P}\}. \end{array}$$

The robot operates in a bounded, obstacle-ridden workspace $𝒲\subset {\mathbb{R}}^{2}$ and is required to navigate from an initial position ${𝐱}_{0}$ to a target region 𝒯⊂𝒲 while avoiding collisions with obstacles. The target is represented as a disk of radius $r_{T}$ centered at $𝐩\in {\mathbb{R}}^{2}$:


$$\begin{array}{c} 𝒯=\{𝐳\in {\mathbb{R}}^{2}:\|𝐳-𝐩\|\leq r_{T}\}. \end{array}$$

Navigation is achieved through a sequence of intermediate waypoints $\{{𝐥}_{k}{\}}_{k=1}^{m}\subset 𝒲$, which act as sub-goals to guide the robot along a feasible, collision-free path from ${𝐱}_{0}$ to 𝐩.

### Kinematic model

The robot is modelled with a system of first-order ordinary differential equations (ODEs). Its velocity is denoted as $𝐮(t)=(u_{1}(t),u_{2}(t))\in {\mathbb{R}}^{2}$. The kinematic equations are therefore


$$\begin{array}{c} \dot{𝐱}(t)=𝐮(t),\;𝐱(0)={𝐱}_{0}, \end{array}$$ (1)

where 𝐱(t) is the robot’s position and 𝐮(t) represents the velocity-based control input.

### Navigation objective

The robot is required to reach the target region 𝒯 via a sequence of intermediate waypoints


$$\begin{array}{c} ℒ=\{{𝐥}_{k}\in {\mathbb{R}}^{2}{\}}_{k=1}^{m}, \end{array}$$

with boundary conditions


$$\begin{array}{c} {𝐥}_{0}={𝐱}_{0},\;{𝐥}_{m+1}=𝐩. \end{array}$$

Each waypoint represents a transient intermediate goal.

### Obstacle model

The workspace contains *q* circular obstacles of arbitrary sizes. Each obstacle l∈{1,...,q} is modeled as a closed disk with center ${𝐨}_{l}\in {\mathbb{R}}^{2}$ and radius $ro_{l}>0$. The obstacle region is defined as


$$\begin{array}{c} FO_{l}=\left\{𝐳\in \mathbb{R}:\|𝐳-{𝐨}_{l}\|\leq ro_{l}\right\}. \end{array}$$

To account for the robot radius $r_{P}$ and a safety margin ϵ>0, define the inflated obstacle region


$$\begin{array}{c} {\tilde{FO}}_{l}=\left\{𝐳\in {\mathbb{R}}^{2}:\|𝐳-{𝐨}_{l}\|\leq ro_{l}+r_{P}+\epsilon \right\}. \end{array}$$

**Assumption 1 (Feasibility).** *The initial and target positions satisfy* ${𝐱}_{0},𝐩\notin \overset{q}{\underset{l=1}{⋃}}{\tilde{FO}}_{l}.$

### Waypoint generation framework

For a fixed number of waypoints *m*, define reference points:


$$\begin{array}{c} {\hat{𝐱}}_{k}={𝐥}_{k-1}+\frac{k}{m+1}(𝐩-{𝐥}_{k-1}),\;k=1,...,m. \end{array}$$

The waypoint generation problem is posed as a non-linear optimization problem:


$$\begin{array}{c} {𝐥}_{k}=\text{arg}\min_{𝐳\in {\mathbb{R}}^{2}}\|𝐳-{\hat{𝐱}}_{k}{\|}^{2} \end{array}$$ (2)

subject to the constraints:


$$\begin{array}{c} \begin{array}{c} dist({𝐨}_{l},[{𝐥}_{k-1},𝐳])\geq ro_{l}+r_{P}+\epsilon ,\;\forall l=1,2,...,q,\\ {𝐥}_{k}\in 𝒲 \end{array}\} \end{array}$$ (3)

where


$$\begin{array}{c} dist(𝐨,[𝐚,𝐛])=\min_{\lambda \in [0,1]}\left\|𝐨-(𝐚+\lambda (𝐛-𝐚))\right\|. \end{array}$$

The optimization problem defined in (2) and (3) is non-convex due to:

- nonlinear distance constraints,
- dependence on line segments,
- obstacle-induced disconnected feasible regions.

Due to its nature, the optimization problem defined in (2) and (3) can be solved numerically using mathematical software such as Wolfram Mathematica or MATLAB. In particular, the Mathematica command

NMinimize[{cost, cons}, vars, Method - > “DifferentialEvolution”]

can be used to efficiently compute a numerical solution of the optimization problem.

The waypoint generation strategy described above is referred to as the Fixed-Horizon Waypoint Generation (FHWG) method, since the reference points ${\hat{𝐱}}_{k}$ are generated using a fixed linear interpolation horizon between the initial and target configurations. The overall procedure is summarized in Algorithm 1.

**Algorithm 1: Fixed-Horizon Waypoint Generation**



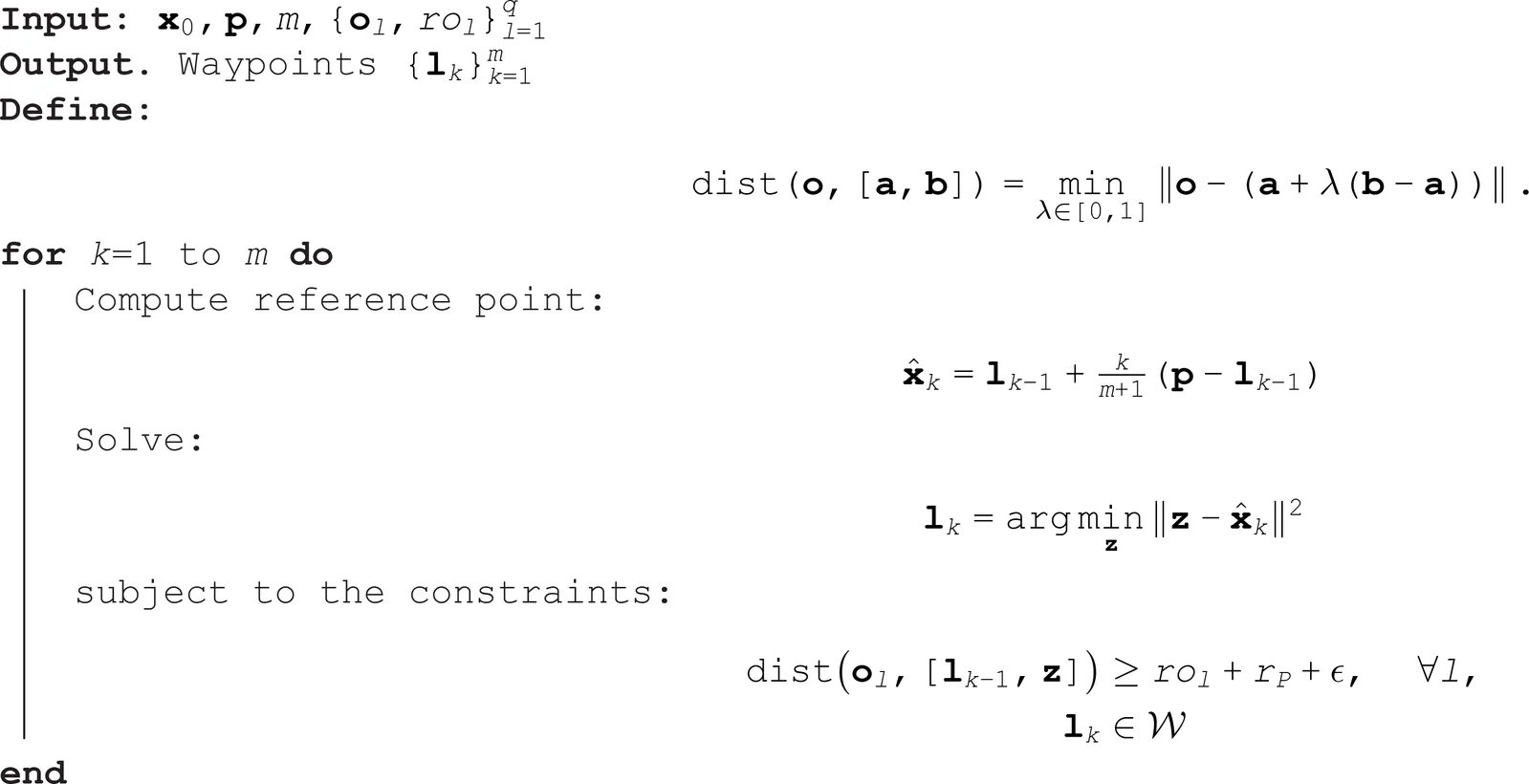



### Waypoint tracking control law

To guide the robot towards the target through the generated waypoints, we adopt a switched Lyapunov-based controller [29]:


$$\begin{array}{c} 𝐮(t)=\alpha \sum_{k=1}^{m+1}\rho_{k}({𝐥}_{k}-𝐱(t)) \end{array}$$ (4)

where α>0 is called the convergence parameter. The switching signal $\rho_{k}\in \{0,1\}$ activates the current waypoint to ensure sequential waypoint tracking:


$$\begin{array}{c} \rho_{k}=\left\{ \begin{array}{cc} 1, & \text{if }\left\|{𝐥}_{k-1}-𝐱(0)\right\|\leq \left\|𝐱-𝐱(0)\right\|<\left\|{𝐥}_{k}-𝐱(0)\right\|;\\ 0, & \text{otherwise.} \end{array}\right. \end{array}$$

This switching mechanism ensures sequential navigation through waypoints, where only the relevant waypoint influences the robot motion at any given time. The effectiveness of the proposed method and its comparison to the APF method is illustrated in Simulation 1.

### Simulation 1

The effectiveness of the proposed waypoint-generation strategy is evaluated in workspaces containing single and multiple circular obstacles. The values of various parameters used in the simulations are given in Table 1.

**Table 1:** Parameters used for the proposed method and APF baseline simulations.

Parameter	Proposed method	APF
Attractive gain, α	0.1	0.1
Repulsive gain, $\beta_{i}$	–	0.1
Integration step, *h*	0.01	0.01
Stopping tolerance	0.005 m	0.005 m
Safety margin, ϵ	0.5 m	0.5 m
Obstacle configuration	Random	Random

In the first scenario shown in Fig 2, the proposed algorithm was evaluated in a workspace containing a single circular obstacle centered at (15,15) with radius 2. The point-mass robot is required to move from the initial position (5,5) to the target position (25,25). Fig 2(a) shows the trajectory obtained using the proposed waypoint-based method with *m* = 2 intermediate waypoints, whereas Fig 2(b) illustrates the trajectory generated by the APF method.

**Fig 2:**
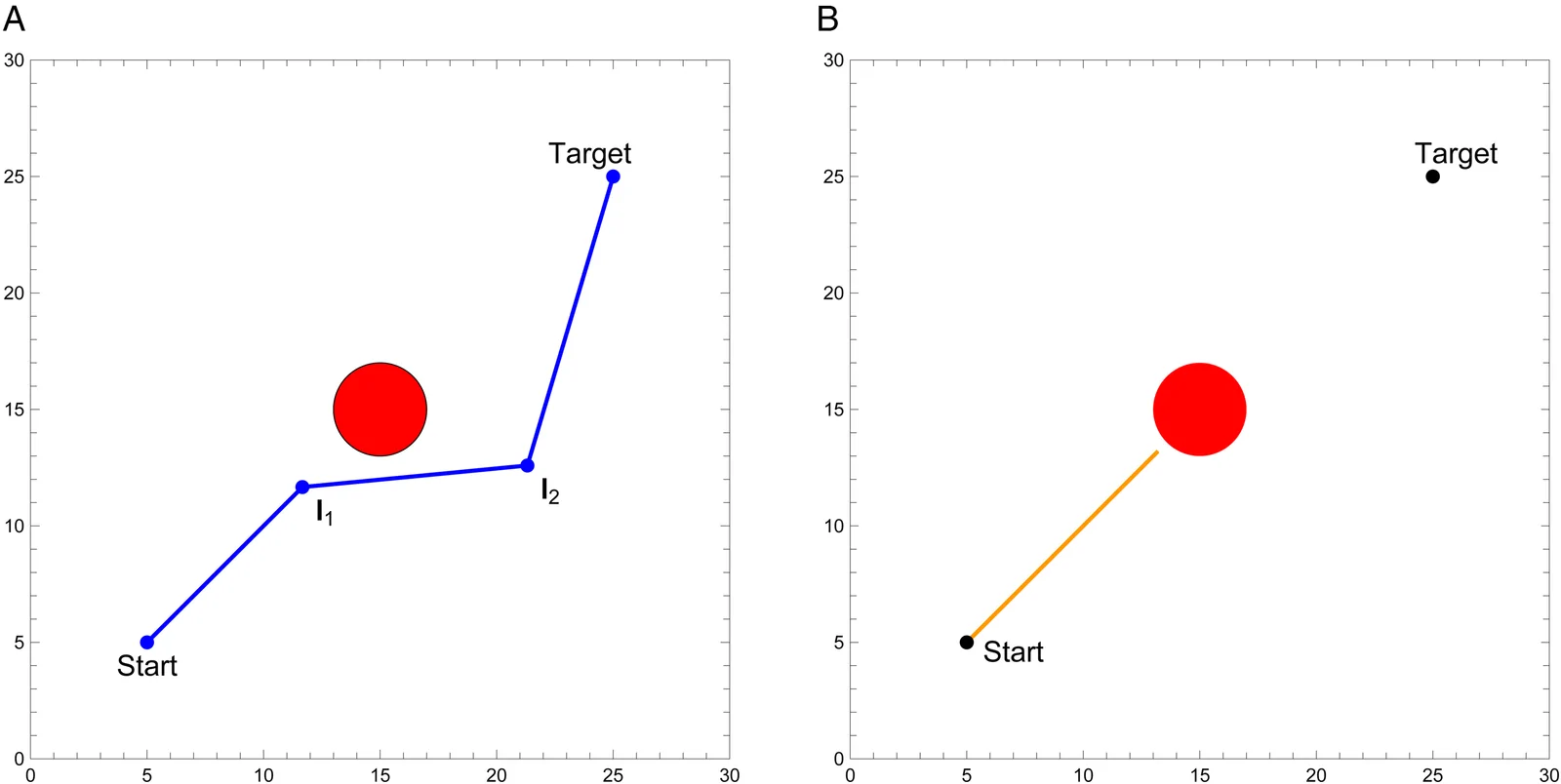
Trajectory of a point-mass robot in the workspace containing one obstacle.

As shown in Fig 2(a), the proposed method successfully generates collision-free intermediate waypoints that guide the robot around the obstacle and toward the target. The generated waypoints serve as temporary goals that progressively steer the robot through obstacle-free regions of the workspace. As a result, the robot maintains continuous progress toward the target while avoiding collision with the obstacle.

In contrast, Fig 2(b) demonstrates a well-known limitation of the APF method. The attractive force toward the target and the repulsive force generated by the obstacle become balanced at a location behind the obstacle, creating a local minimum of the potential field. Consequently, the robot becomes trapped at this equilibrium point and fails to reach the target. This example highlights the ability of the proposed waypoint-generation strategy to overcome local-minimum problems that commonly affect APF-based navigation.

In the second scenario shown in Fig 3, the workspace contains ten circular obstacles with randomly generated positions and radii. The robot starts at (25,5) and is required to reach the target located at (5,25). The proposed method employs *m* = 3 intermediate waypoints to generate a feasible path through the obstacle-free region. As shown in Fig 3(a), the resulting trajectory is collision-free and successfully guides the robot to the target. Fig 3(b) shows the trajectory obtained using the APF method. Unlike the previous scenario, the APF method is also able to reach the target without becoming trapped in a local minimum.

**Fig 3:**
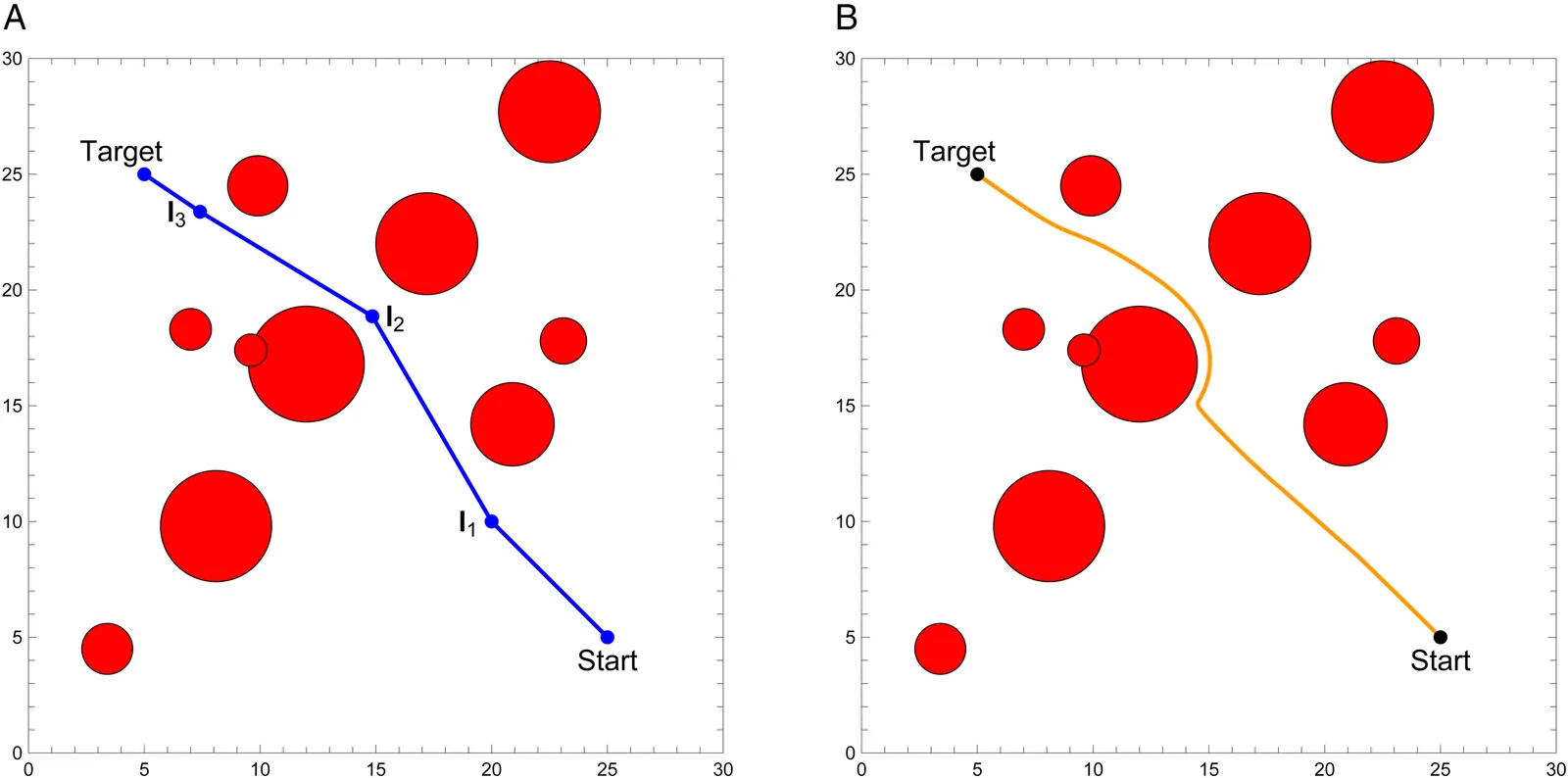
Trajectory of a point-mass robot in the workspace containing multiple obstacles.

To further compare the two approaches, the total path length and the time required to reach the target were evaluated for the simulation presented in Fig 3. The proposed method produced a path length of 28.93 units and a travel time of 287.06 units, whereas the APF method generated a path length of 29.93 units and a travel time of 87.61 units.

These results reveal an interesting trade-off. The waypoint-based method generates a slightly shorter path, reducing the total travel distance by approximately 3.2% compared with the APF method. The shorter path is a consequence of the optimization process, which explicitly selects intermediate waypoints that lie close to the straight-line route between the start and target while satisfying obstacle-avoidance constraints. However, despite the shorter path length, the travel time is significantly larger than that of the APF method. This behavior arises because the robot slows down as it approaches each waypoint before proceeding to the next one, resulting in a larger cumulative travel time. In contrast, the APF method continuously generates a control action directed toward the target, allowing the robot to maintain a more direct and responsive motion and thereby reach the target in a shorter time.

Overall, the results demonstrate that the proposed waypoint-based method provides reliable collision-free navigation and effectively avoids the local-minimum problem associated with APF methods. Furthermore, it can produce paths that are slightly shorter than those generated by APF. The trade-off is an increase in travel time due to the sequential convergence of the robot to intermediate waypoints.

The Fixed-Horizon Waypoint Generation method is essentially a greedy, sequential waypoint optimization scheme in which each waypoint is generated based only on the previous point and a moving reference segment toward the goal, while satisfying non-convex segment–obstacle clearance constraints through numerical optimization. Although this formulation can provide local collision avoidance, it is fundamentally non-convex and does not provide global guarantees of optimality or reliable convergence to the target in complex environments. The reliance on segment-based distance constraints also introduces non-smoothness and numerical sensitivity, while the use of heuristic solvers such as Differential Evolution may lead to solver-dependent performance, particularly in cluttered environments or narrow-passage scenarios.

Fig 4 illustrates a representative failure case in which the robot is unable to reach the target due to a concave arrangement of circular obstacles forming a dead-end region. As shown in Fig 4(a), the proposed method produces a non-reachable trajectory that becomes trapped within the obstacle configuration. A similar failure is observed for the Artificial Potential Field (APF) method in Fig 4(b), where the robot is also unable to escape the local minimum induced by the concave obstacle structure.

**Fig 4:**
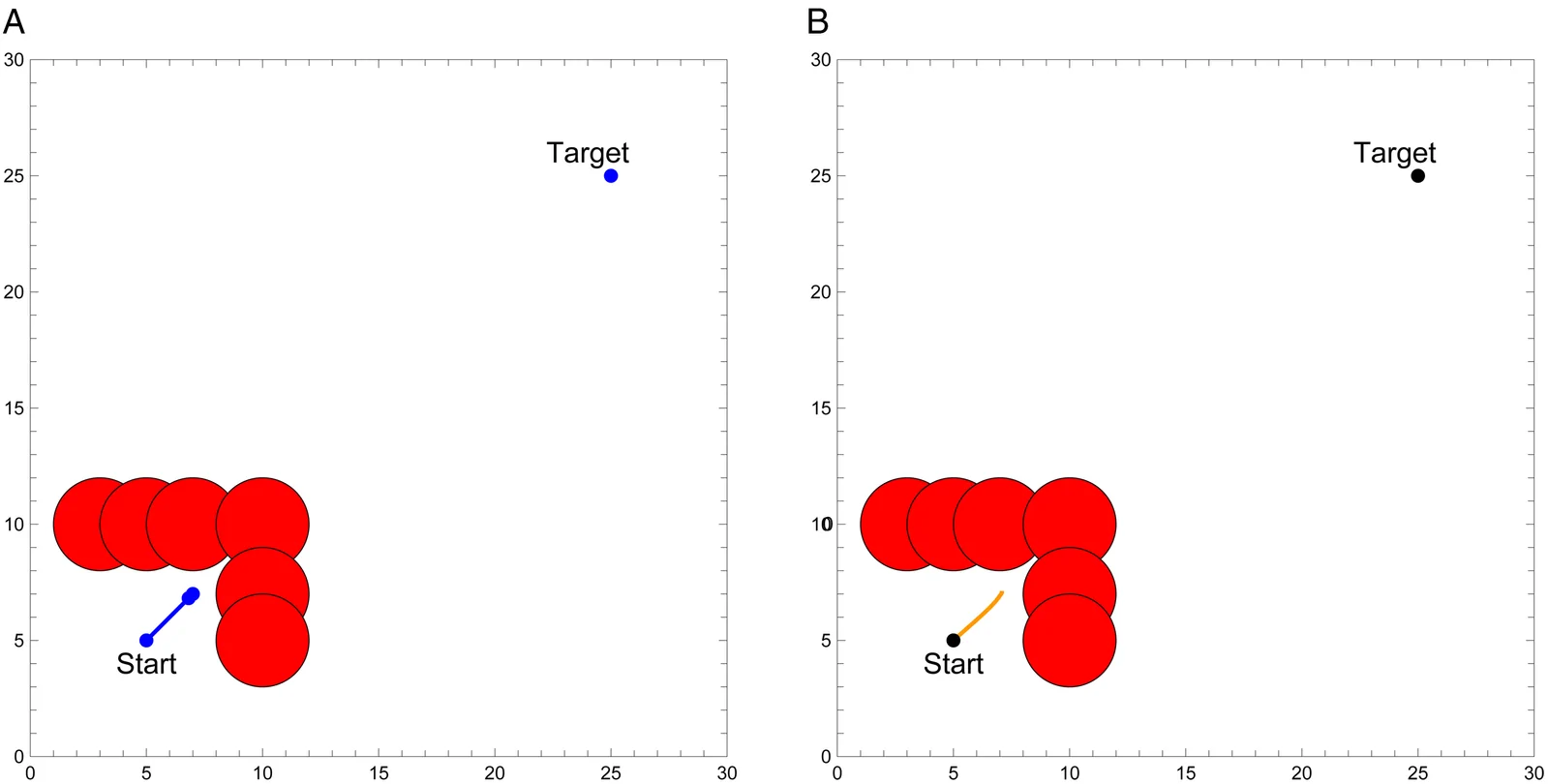
Failure case in a concave obstacle environment. The concave obstacle arrangement creates a dead-end region that prevents convergence to the target.

From simulations, it is observed that Algorithm 1 performs effectively in scenarios with simple obstacle configurations and a pre-determined number of waypoints. However, its performance becomes increasingly dependent on the pre-specified number of waypoints *m*. In particular, an inappropriate choice of *m* may lead to either unnecessary computational overhead (when *m* is too large) or insufficient obstacle avoidance capability (when *m* is too small), potentially compromising path feasibility in cluttered environments. To further quantify this dependence, a sensitivity analysis with respect to the waypoint horizon and numerical solver settings is presented below.

### Sensitivity analysis of the Fixed-Horizon formulation

The Fixed-Horizon Waypoint Generation formulation requires the number of intermediate waypoints to be priori specified. Moreover, each waypoint is obtained by solving a non-convex constrained optimization problem using the Differential Evolution method implemented through NMinimize. Consequently, both the prescribed waypoint horizon and the stochastic numerical optimization procedure can affect the computational cost and the obtained solution. To quantify these effects, a sensitivity analysis is performed by varying the waypoint count while keeping the planning problem and solver configuration otherwise unchanged.

For each selected value of


$$\begin{array}{c} m\in \{1,2,...,10\}, \end{array}$$

the start position, target position, obstacle configuration, robot parameters, safety margin, and optimization formulation were kept unchanged. The test environment used in this analysis is shown in Fig 5.

**Fig 5:**
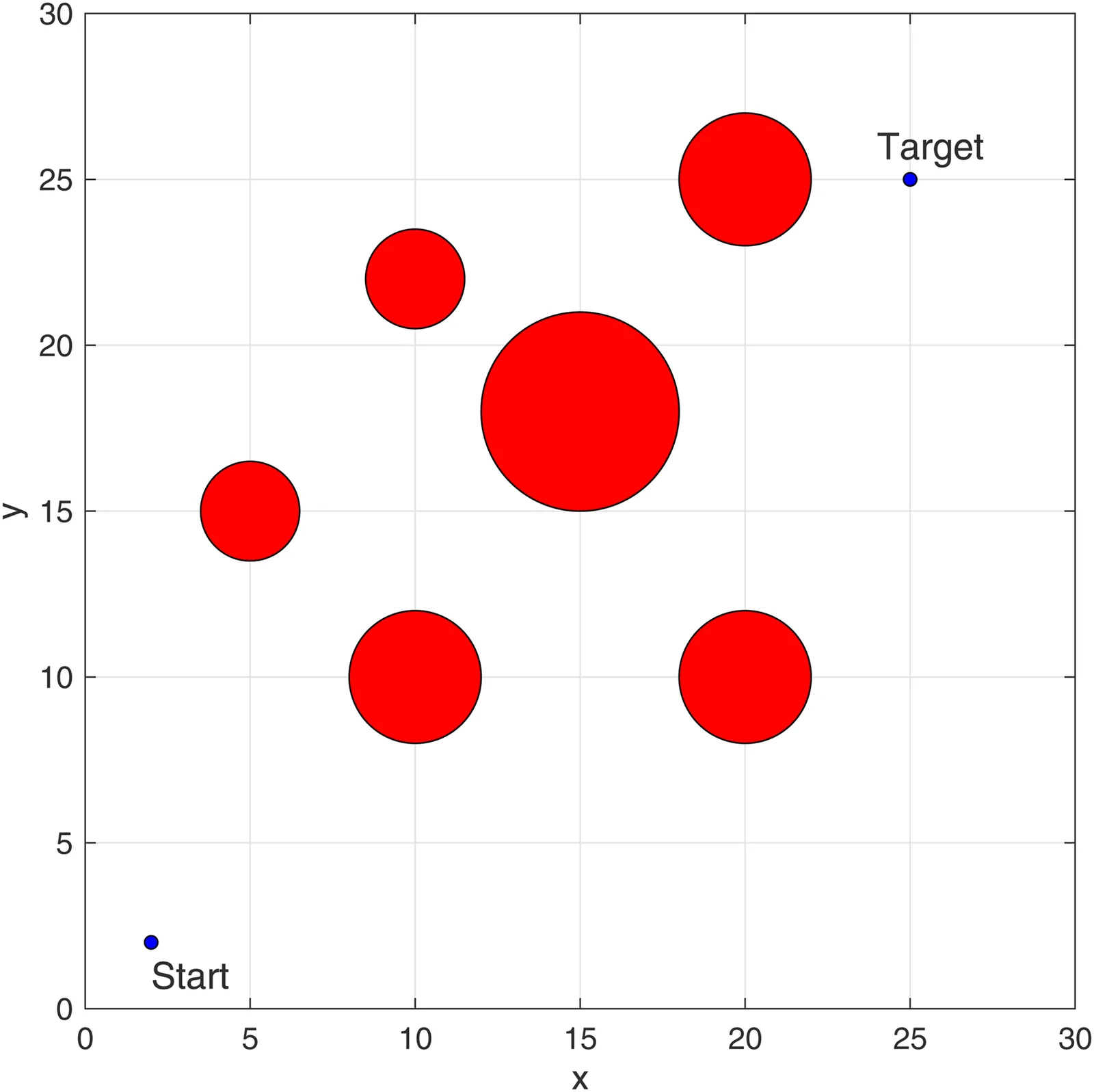
The test environment.

Ten independent runs of the Differential Evolution solver were performed for each value of *m*, using different random seeds. Table 2 summarizes the resulting feasibility, total motion time, and total path length.

**Table 2:** Sensitivity of the Fixed-Horizon formulation to the prescribed number of intermediate waypoints. Results are based on 10 independent differential evolution runs for each value of *m.*

Waypoint count *m*	Feasibility (%)	Total motion time (s)	Total path length (m)
1	100	293.2±0.0	33.6±0.00
2	0	–	–
3	100	293.2±0.0	35.6±0.00
4	100	353.2±0.0	34.9±0.00
5	100	403.2±0.0	33.9±0.00
6	100	451.0±0.0	33.8±0.00
7	0	–	–
8	100	495.0±0.0	40.4±0.00
9	100	461.6±0.0	43.5±0.00
10	100	565.6±0.0	42.7±0.00

The results demonstrate that the feasibility of the Fixed-Horizon formulation depends on the prescribed waypoint count and is not monotonic with respect to *m*. For the considered environment, a feasible solution is obtained with *m* = 1, whereas *m* = 2 is infeasible. Feasible solutions are obtained again for *m* = 3–6, while *m* = 7 is infeasible. Feasibility is recovered for *m* = 8–10. This behavior indicates that increasing the number of prescribed waypoints does not necessarily improve feasibility. The reason is that each additional waypoint introduces another constrained optimization stage and modifies the recursive target used in subsequent waypoint-generation steps. Consequently, a larger waypoint count can alter the sequence of intermediate optimization problems in a way that may either facilitate or prevent the generation of a feasible path.

The trajectory performance also varies with the prescribed waypoint count. Among the feasible cases, the total path length ranges from approximately 33.6 m to 43.5 m, while the total motion time ranges from approximately 293.2 s to 565.6 s. In particular, increasing *m* does not consistently reduce either path length or motion time. For example, the shortest path among the tested feasible cases is obtained for *m* = 1, with a path length of approximately 33.6 m. A similarly short path is obtained for *m* = 6, with a path length of approximately 33.8 m. However, increasing the waypoint count does not consistently reduce the resulting path length, as demonstrated by the substantially longer paths obtained for *m* = 8–10. Similarly, the total motion time increases considerably for larger values of *m*, reaching approximately 565.6 s for *m* = 10.

For the present environment, the ten independent Differential Evolution runs produced numerically identical path lengths and motion times for each feasible value of *m*, resulting in zero standard deviation. This observation indicates that the solver repeatedly converged to the same numerical solution for this particular test case. However, the zero standard deviation should not be interpreted as a general guarantee of deterministic behavior, since Differential Evolution is inherently a stochastic optimization method.

Overall, the sensitivity analysis confirms that the Fixed-Horizon formulation is dependent on the user-prescribed waypoint count. An inappropriate value of *m* may result in infeasibility, while increasing *m* does not necessarily improve the resulting trajectory and can increase the associated motion time. These observations highlight a limitation of the Fixed-Horizon formulation and motivate the adaptive waypoint-generation strategy developed in the following section, in which waypoints are generated incrementally only when required by obstacle interference, thereby eliminating the need to prescribe the total number of intermediate waypoints in advance.

To address the limitations of the Fixed-Horizon Waypoint Generation (FHWG) method, we develop an adaptive strategy that separates waypoint generation from continuous non-convex optimization. Instead of solving a new non-convex optimization problem for every waypoint, the proposed Adaptive Waypoint Generation with Collision Checking framework evaluates a prescribed finite set of candidate points and explicitly checks the collision-free feasibility of the corresponding path segments. A new waypoint is generated only when the direct segment toward the goal is obstructed. This produces an incremental planning procedure in which segment-wise feasibility is explicitly verified at each waypoint-selection step.

## The adaptive waypoint generation scheme

We now improve upon the fixed-horizon waypoint optimization framework by adaptively generating waypoints only when necessary, based on segment-wise collision checks. We also remove the dependence on a fixed number of waypoints.

### Segment feasibility test

At each iteration *k*, the direct connection from the current waypoint ${𝐥}_{k-1}$ to the goal 𝐩 is evaluated using the minimum distance from each obstacle to the segment $\left[{𝐥}_{k-1},𝐩\right]$. The segment is collision-free if


$$\begin{array}{c} dist({𝐨}_{l},[{𝐥}_{k-1},𝐩])\geq ro_{l}+r_{P}+\epsilon ,\;\forall l. \end{array}$$

If this condition holds, the goal is reachable directly, and the algorithm terminates with ${𝐥}_{k}=𝐩$.

### Adaptive waypoint construction

If the segment $\left[{𝐥}_{k-1},𝐩\right]$ is obstructed, define the set of obstacles intersecting the clearance margin:


$$\begin{array}{c} {ℐ}_{k}=\{l|dist({𝐨}_{l},[{𝐥}_{k-1},𝐩])<ro_{l}+r_{P}+\epsilon \}. \end{array}$$

A heuristic guidance point ${\hat{𝐱}}_{k}$ is constructed using the centroid of these obstacles:


$$\begin{array}{c} {\bar{𝐨}}_{k}=\frac{1}{\left|{ℐ}_{k}\right|}\sum_{l\in {ℐ}_{k}}{𝐨}_{l}. \end{array}$$

The reference point is then defined as


$$\begin{array}{c} {\hat{𝐱}}_{k}={𝐥}_{k-1}+\lambda_{k}^{*}(𝐩-{𝐥}_{k-1}), \end{array}$$

where


$$\begin{array}{c} \lambda_{k}^{*}=\min\left\{1,\max\left\{0,\frac{\left({\bar{𝐨}}_{k}-{𝐥}_{k-1}cdot(𝐩-{𝐥}_{k-1}\right)}{\|𝐩-{𝐥}_{k-1}{\|}^{2}}\right\}\right\} \end{array}$$

The next waypoint ${𝐥}_{k}$ is then obtained by solving:


$$\begin{array}{c} {𝐥}_{k}=\text{arg}\min_{𝐳\in {\mathbb{R}}^{2}}\|𝐳-{\hat{𝐱}}_{k}{\|}^{2} \end{array}$$ (5)

subject to the constraints:


$$\begin{array}{c} dist({𝐨}_{l},[{𝐥}_{k-1},𝐳])>ro_{l}+r_{P}+\epsilon ,\;\forall l, \end{array}$$ (6)

$$\begin{array}{c} dist({𝐨}_{l},[𝐳,𝐳+\gamma (𝐩-𝐳)])>ro_{l}+r_{P}+\epsilon ,\;\forall l, \end{array}$$ (7)

$$\begin{array}{c} \|z-p\|<\|l_{k-1}-p\|, \end{array}$$ (8)

$$\begin{array}{c} {𝐥}_{k}\in 𝒲 \end{array}$$ (9)

where 0<γ≤1 defines a lookahead portion toward the goal. The lookahead constraint (7) provides a heuristic safeguard against immediate blockage by concave or tightly clustered obstacles. This ensures that locally feasible waypoints do not trap the path in regions where a direct segment to the goal would be obstructed. Moreover, the strict condition 8 prevents any candidate from being selected more than once and ensures forward progress towards the target. The adaptive framework also eliminates dependence on a pre-specified number of waypoints *m*, enforces segment-wise collision avoidance, and maintains forward progress toward the goal.

### Numerical solution

To reduce computational complexity in dense environments, we propose a discretization-based approximation of the continuous optimization problem in (5)–(9).The workspace is discretized into a finite candidate set $ℱ=\{{𝐟}_{i}{\}}_{i=1}^{n}$. At each iteration, only candidate points satisfying


$$\begin{array}{c} dist({𝐨}_{l},[{𝐥}_{k-1},{𝐟}_{i}])>ro_{l}+r_{P}+\epsilon \end{array}$$

and


$$\begin{array}{c} dist({𝐨}_{l},[{𝐟}_{i},{𝐟}_{i}+\gamma (𝐩-{𝐟}_{i})])>ro_{l}+r_{P}+\epsilon \end{array}$$

and


$$\begin{array}{c} \left\|f_{i}-p\|<\|l_{k-1}-p\right\| \end{array}$$

are considered feasible. Among all feasible candidate points, the point closest to ${\hat{x}}_{k}$ is selected as next waypoint ${𝐥}_{k}$. Equivalently, the waypoint is chosen as


$$\begin{array}{c} {𝐥}_{k}=\text{arg}\min_{{𝐟}_{i}\in F_{k}^{feas}}D_{i,k}. \end{array}$$

where


$$\begin{array}{c} D_{i,k}=\left\{ \begin{array}{cc} \infty , & \exists \;l:dist({𝐨}_{l},[{𝐥}_{k-1},{𝐟}_{i}]lero_{l}+r_{P}+\epsilon \\ & \text{ or }dist({𝐨}_{l},[{𝐟}_{i},{𝐟}_{i}+\gamma (𝐩-{𝐟}_{i})])\leq ro_{l}+r_{P}+\epsilon \\ \|f_{i}-{\hat{x}}_{k}{\|}^{2}, & \text{otherwise} \end{array}\right. \end{array}$$

and


$$\begin{array}{c} F_{k}^{feas}=\left\{f_{i}\in F|D_{i,k}<\infty ,\;\|f_{i}-p\|<\|l_{k-1}-p\|\right\} \end{array}$$

Unlike the Fixed-Horizon formulation, which relies on repeated numerical optimization of a non-convex problem, the proposed adaptive strategy uses a prescribed finite candidate set $ℱ=\{{𝐟}_{i}{\}}_{i=1}^{n}$. When the direct segment from the current waypoint to the target is obstructed, the algorithm evaluates each candidate according to its collision-free feasibility and its distance to the corresponding moving reference point. Infeasible candidates are discarded, and the feasible candidate with the minimum selection cost is chosen as the next waypoint. Consequently, the adaptive candidate-selection stage does not rely on stochastic sampling or repeated non-convex optimization. Since ℱ is finite, the candidate evaluation at each waypoint-selection step is also finite.

The centroid of the currently blocking obstacles is used to construct the local reference point ${\hat{𝐱}}_{k}$, which guides the selection of the next waypoint. Importantly, ${\hat{𝐱}}_{k}$ does not define or restrict the candidate search region. The candidate set $ℱ=\{{𝐟}_{i}{\}}_{i=1}^{n}$ is generated over the workspace, and each candidate is evaluated according to the collision-free connectivity conditions in Algorithm 2. Specifically, a candidate ${𝐟}_{i}$ is considered feasible only if the segment from the current waypoint ${𝐥}_{k-1}$ to ${𝐟}_{i}$ is collision-free and the lookahead segment from ${𝐟}_{i}$ toward the target is also collision-free. In addition, the candidate must provide progress toward the target, i.e.,


$$\begin{array}{c} \|{𝐟}_{i}-𝐩\|<\|{𝐥}_{k-1}-𝐩\|. \end{array}$$ (10)

The next waypoint is then selected as the feasible candidate that minimizes the distance to the centroid-based reference point.

Consequently, in an asymmetric obstacle configuration, the centroid may be biased toward one side of the blocking obstacles, thereby influencing the preferred location of the next waypoint. However, it does not prevent feasible candidates elsewhere in the workspace from being considered. Nevertheless, the centroid remains a geometric heuristic and does not explicitly encode the topology of the free space. In particular, for highly asymmetric corridors or environments containing disconnected free-space components, the centroid-based reference may not provide an optimal representation of the available free-space structure. The proposed method therefore does not claim globally optimal behavior for arbitrary disconnected environments. Instead, its objective is to provide a computationally efficient local waypoint-selection mechanism based on obstacle geometry and collision-free connectivity.

The Adaptive Waypoint Generation scheme is summarised in Algorithm 2.

**Algorithm 2: Adaptive waypoint generation with collision checking.**



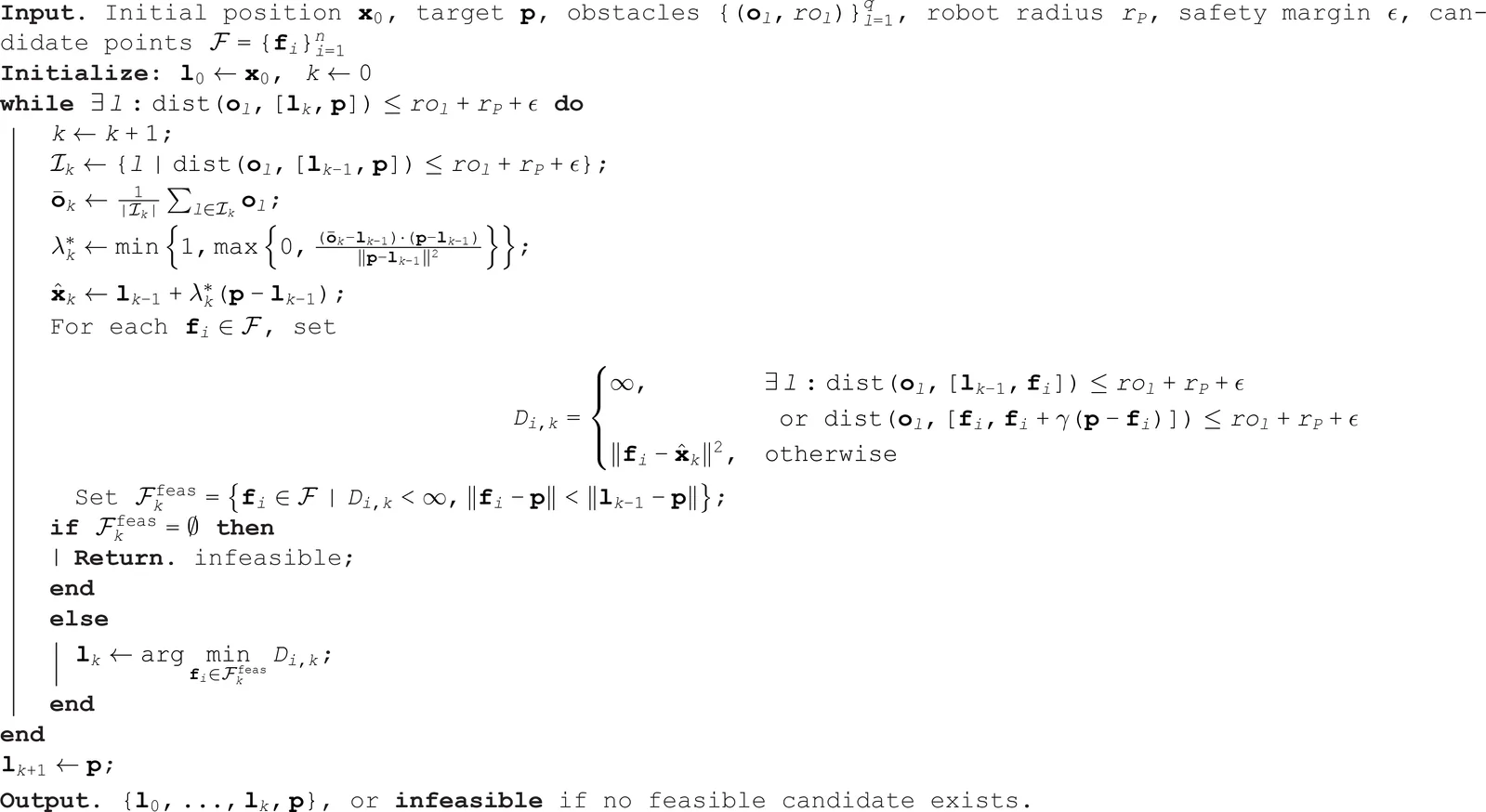



Compared to classical fixed-waypoint schemes, the proposed method exhibits the following properties:

- **Adaptive**: The number of waypoints is determined online based on obstacle configuration rather than a pre-specified discretization parameter.
- **Segment-aware**: Collision avoidance is enforced over continuous line segments connecting waypoints, rather than only at discrete sampled points.
- **Computationally efficient**: The use of localized optimization and discrete candidate sampling reduces computational overhead compared to global search methods.
- **Practically robust**: While global completeness may not be guaranteed in arbitrarily complex environments, the method performs well in simple to moderately cluttered scenarios, including environments with narrow passages.

To guide the robot through the generated waypoints, we define the waypoint set $𝒮=\{{𝐥}_{0},{𝐥}_{1},...,{𝐥}_{N}\}$, where ${𝐥}_{N}=𝐩$. A switched Lyapunov-based controller is adopted:


$$\begin{array}{c} 𝐮(t)=\alpha ({𝐥}_{i(t)}-𝐱(t)), \end{array}$$ (11)

where α>0 is the convergence parameter and


$$\begin{array}{c} i(t)=\left\{k\in \{1,...,N\}\;|\;\left\|{𝐥}_{k-1}-𝐱(0)\right\|\leq \left\|𝐱(t)-𝐱(0)\right\|<\left\|{𝐥}_{k}-𝐱(0)\right\|\right\} \end{array}$$

denotes the active waypoint index.

### Simulation 2

Fig 6 illustrates the effectiveness of the proposed method in overcoming the local-minimum problem caused by the concave obstacle arrangement. Unlike the fixed-horizon waypoint generation and APF methods, which become trapped within the dead-end region, the proposed strategy generates an intermediate waypoint ${𝐈}_{1}$ that lies in a reachable free-space area and provides a viable escape direction from the obstacle configuration. Consequently, the robot is able to safely bypass the obstacle cluster and continue toward the target. The generated waypoint preserves obstacle avoidance, local minima trap avoidance and goal-directed motion, resulting in successful navigation to the destination. Fig 7 presents a navigation scenario involving 15 obstacles of random sizes and positions. Both the proposed method and the APF method successfully reach the target without collision. The proposed approach generates two intermediate waypoints that guide the robot through the free-space regions, resulting in a path length of 31.689 units. In comparison, the APF method produces a longer trajectory of 33.574 units. Although the proposed method yields a shorter and potentially more efficient path, the corresponding time-to-goal is 268.9s, whereas the APF method reaches the target in only 92.4 s. Therefore, the proposed method demonstrates superior path efficiency, while the APF method achieves faster target attainment in this particular scenario.

**Fig 6:**
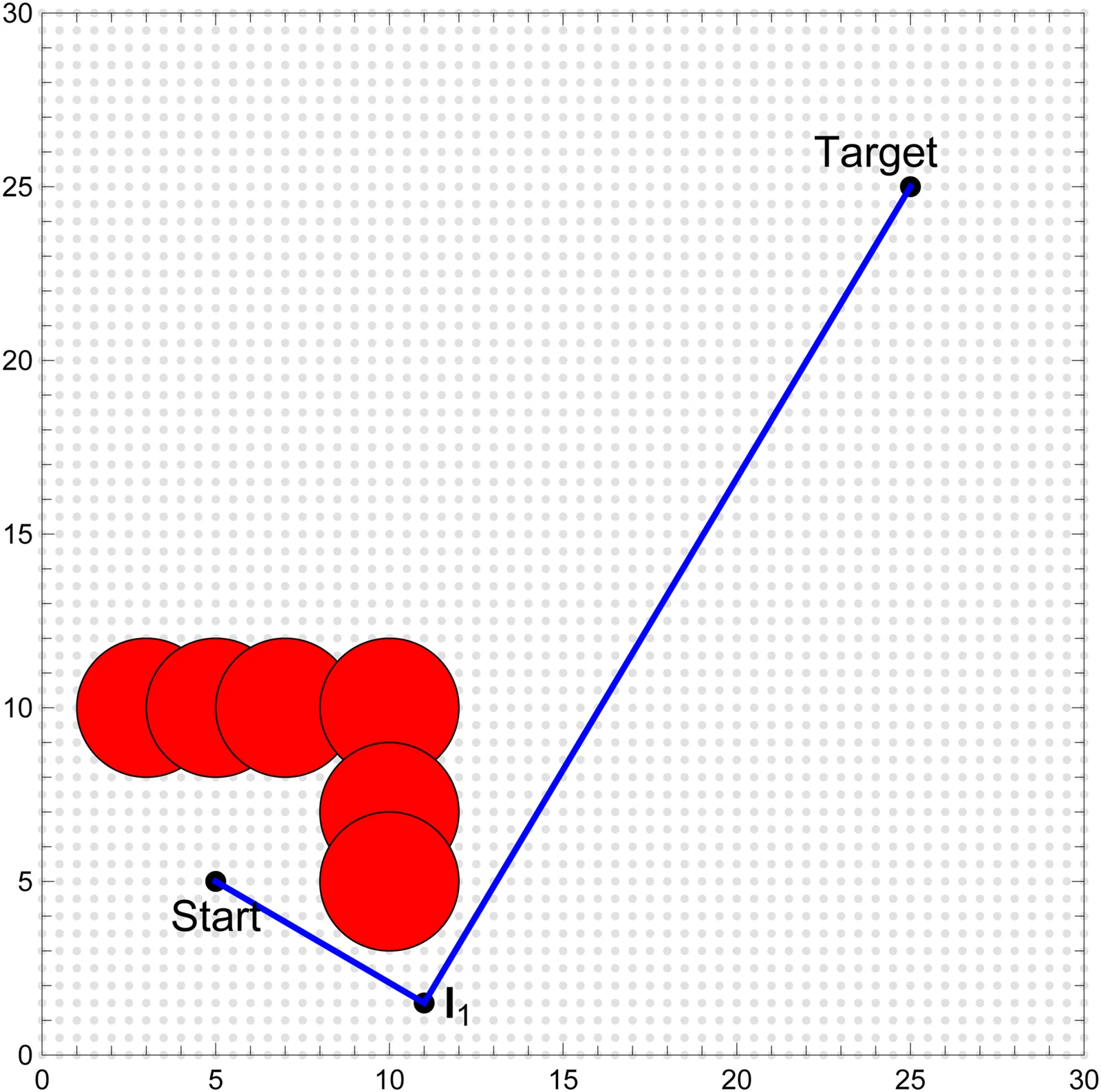
Performance of the proposed adaptive waypoint generation scheme in the dead-end obstacle environment. The 6 obstacles form a concave arrangement.

**Fig 7:**
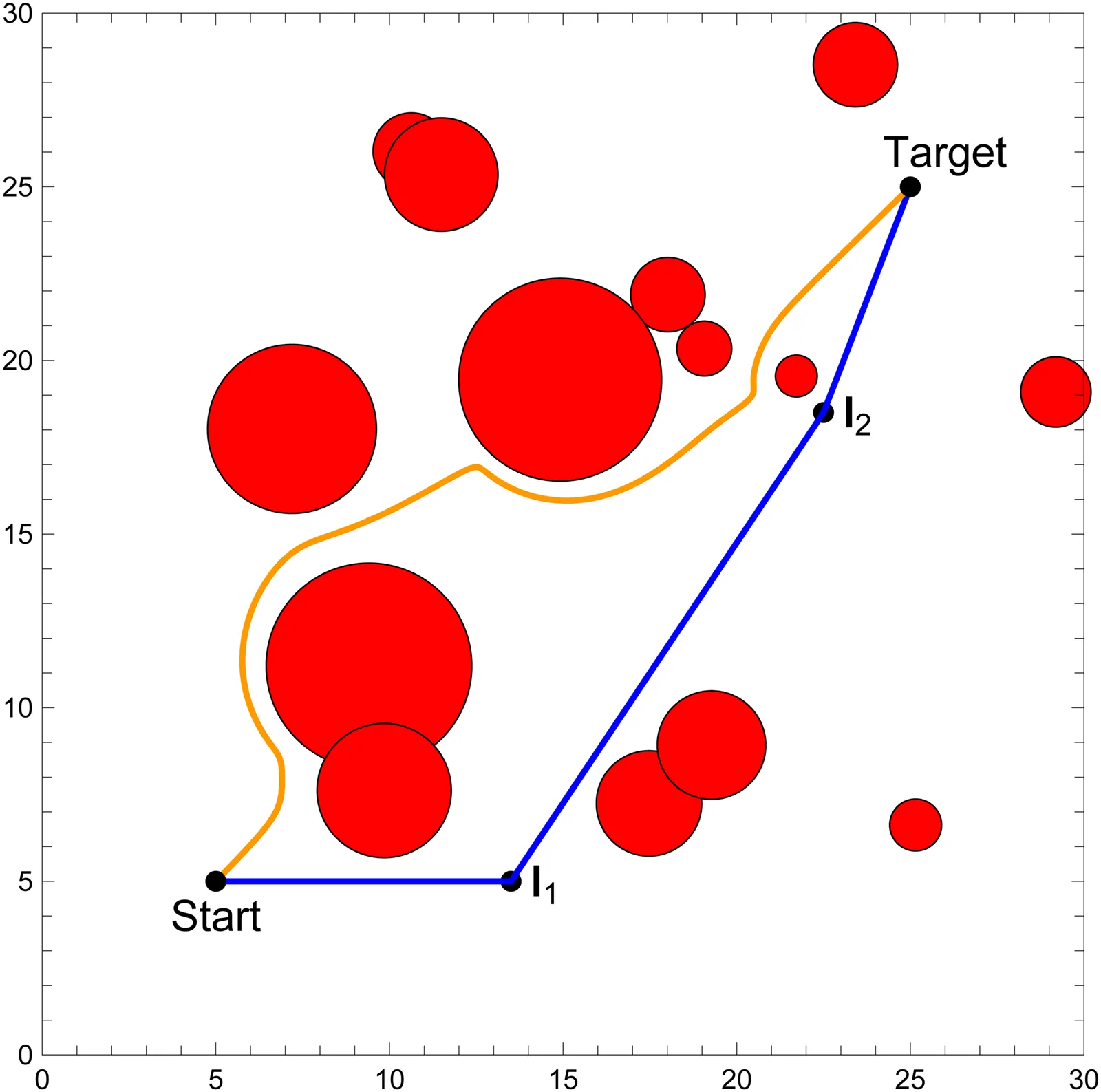
Navigation trajectories in a workspace containing 15 obstacles of random sizes and positions. Blue – proposed method, brown – APF method.

Fig 8 illustrates the navigation performance in an environment containing 30 randomly distributed obstacles with varying sizes. In this scenario, the proposed Adaptive Waypoint Generation (AWG) scheme successfully guides the robot to the target by generating three intermediate waypoints. These waypoints enable the robot to progressively navigate through the cluttered environment while avoiding obstacle-induced local minima. In contrast, the APF method becomes trapped within the obstacle field due to the presence of competing attractive and repulsive forces, preventing successful target attainment. The results demonstrate the ability of the proposed method to adaptively generate reachable intermediate goals and maintain progress toward the target in moderately dense environments. Fig 9 presents a more challenging environment containing 50 randomly positioned obstacles of varying sizes. Owing to the increased obstacle density and complexity of the free-space topology, the proposed AWG scheme generates five intermediate waypoints to safely guide the robot toward the target. By adaptively introducing additional waypoints when necessary, the method successfully avoids local minima and identifies feasible navigation corridors through the cluttered workspace. Conversely, the APF method becomes trapped before reaching the target, illustrating its susceptibility to local-minimum conditions in highly congested environments. These results highlight the robustness and adaptability of the proposed approach as environmental complexity increases.

**Fig 8:**
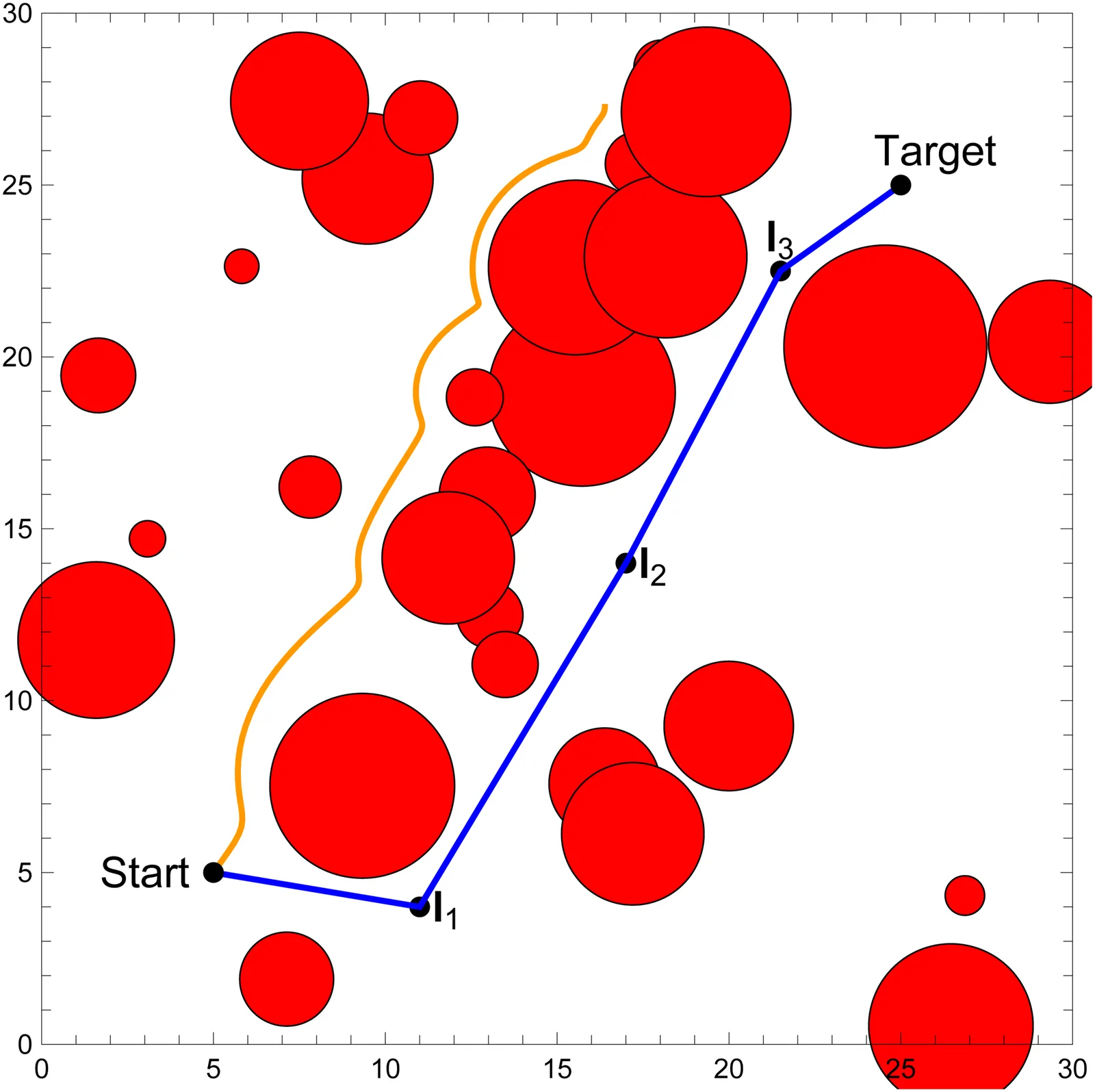
Workspace containing 30 randomly distributed obstacles. Blue path – proposed method converging to target, brown path – APF method leading to local minima trap.

**Fig 9:**
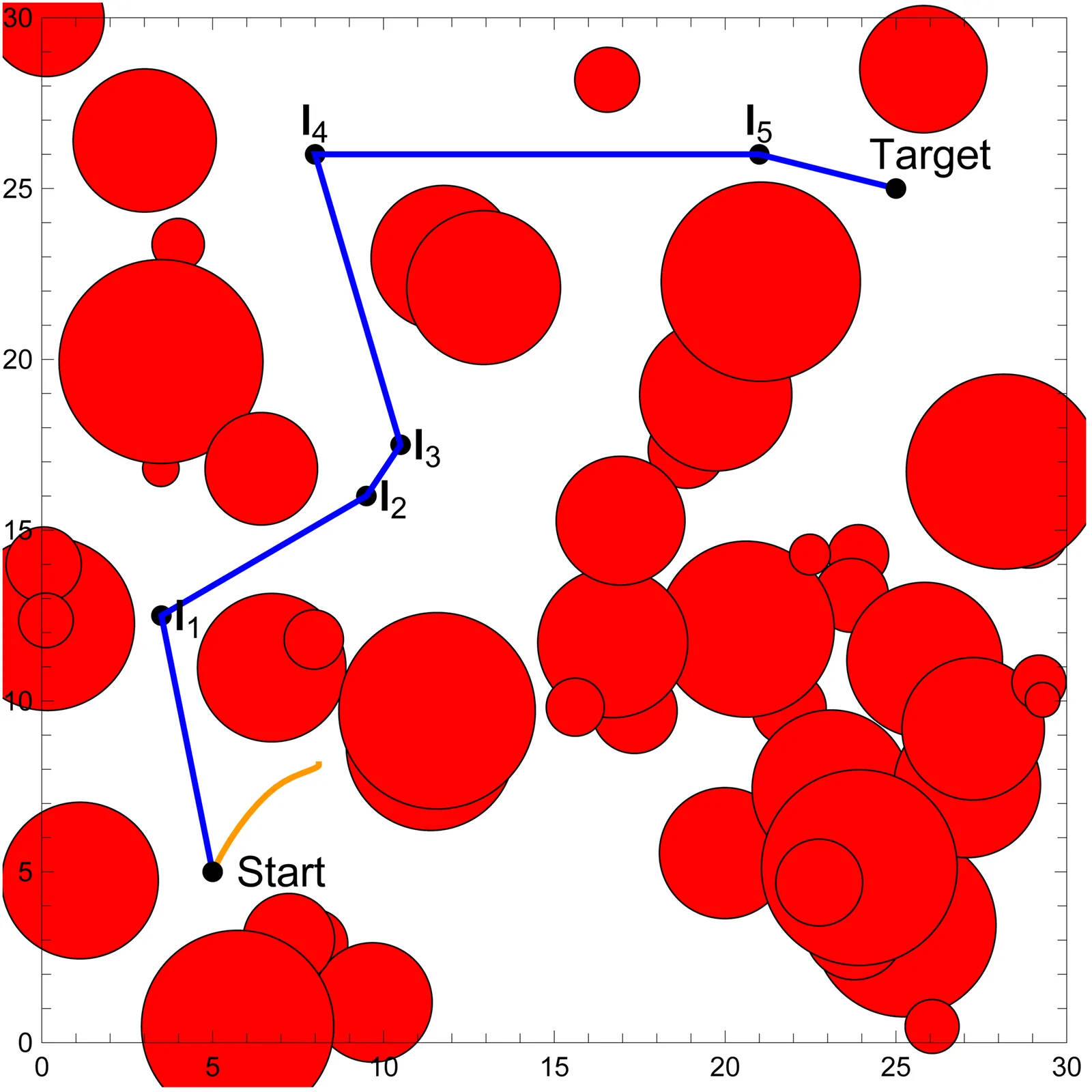
Workspace containing 50 randomly distributed obstacles. Blue path – proposed method converging to target, brown path – APF method leading to local minima trap.

The results obtained in the 30- and 50-obstacle environments demonstrate the scalability of the proposed AWG scheme. As the complexity of the environment increases, the method automatically generates additional intermediate waypoints to maintain a feasible path toward the target. Specifically, three waypoints were required in the 30-obstacle scenario, while five waypoints were generated in the 50-obstacle scenario. This adaptive behavior enables the robot to avoid obstacle-induced local minima and successfully reach the target in environments where the APF method fails. The findings suggest that the proposed approach provides a robust navigation framework capable of handling increasingly cluttered and complex obstacle configurations without requiring brute-force parameter adjustment or prior knowledge of the entire workspace.

Overall, the simulation results demonstrate that the proposed Adaptive Waypoint Generation strategy effectively addresses one of the major shortcomings of the APF method. Even in environments where the APF method fails completely, the proposed method is capable of generating feasible navigation paths and guiding the robot safely to its destination.

**Proposition 1 (Finite termination of the waypoint-generation procedure).** *Assume that the candidate set* $ℱ=\{{𝐟}_{i}{\}}_{i=1}^{n}$ *is finite and that a candidate waypoint is selected only if it satisfies the collision-free condition* $D_{i,k}<\infty$ *and the strict progress condition*


$$\begin{array}{c} \|{𝐟}_{i}-𝐩\|<\|{𝐥}_{k-1}-𝐩\|. \end{array}$$ (12)

*Then Algorithm 2 terminates after a finite number of iterations, with at most n waypoint-selection iterations.*

**Proof.** Let


$$\begin{array}{c} d_{k}=\|{𝐥}_{k}-𝐩\| \end{array}$$ (13)

denote the distance from the *k*-th waypoint to the target. By the strict progress condition in (12), every selected waypoint satisfies


$$\begin{array}{c} d_{k}<d_{k-1}. \end{array}$$ (14)

Thus, the sequence $\left\{d_{k}\right\}$ is strictly decreasing.

Consequently, no candidate waypoint can be selected more than once. Indeed, suppose, for contradiction, that ${𝐥}_{j}={𝐥}_{k}$ for some *j* < *k*. Since the waypoint sequence is strictly decreasing in distance to the target, we have


$$\begin{array}{c} d_{k}<d_{j}. \end{array}$$ (15)

However, ${𝐥}_{j}={𝐥}_{k}$ implies


$$\begin{array}{c} d_{k}=\|{𝐥}_{k}-𝐩\|=\|{𝐥}_{j}-𝐩\|=d_{j}, \end{array}$$ (16)

which is a contradiction. Therefore, every selected waypoint is distinct.

Since ℱ contains only *n* candidate waypoints, at most *n* distinct candidate waypoints can be selected. At each iteration, if no candidate satisfies both the collision-free and strict-progress conditions, i.e.,


$$\begin{array}{c} {ℱ}_{k}^{feas}=\emptyset , \end{array}$$ (17)

the algorithm terminates by returning **infeasible**. Otherwise, a new candidate waypoint is selected. Hence, the candidate-search procedure terminates after finitely many iterations, with at most *n* waypoint selections.

Once the direct segment from the final waypoint ${𝐥}_{k}$ to the target 𝐩 is collision-free, the while-condition in Algorithm 2 becomes false, and the algorithm terminates with the finite waypoint sequence


$$\begin{array}{c} \{{𝐥}_{0},...,{𝐥}_{k},𝐩\}. \end{array}$$ (18)

Therefore, Algorithm 2 is guaranteed to terminate in finite time.

### Convergence analysis

**Theorem 1 (Asymptotic convergence to the target).** *Let Assumption 1 hold. Suppose that the initial position* ${𝐱}_{0}$ *and the target position* 𝐩 *belong to the same path-connected component of the clearance-adjusted free space*


$$\begin{array}{c} {𝒳}_{free}={\mathbb{R}}^{2}⧵\overset{q}{\underset{l=1}{⋃}}{\tilde{FO}}_{l}, \end{array}$$

*where* ${\tilde{FO}}_{l}$ *denotes the obstacle region inflated by the robot radius* $r_{P}$ *and safety margin* ϵ*. Furthermore, assume that for every waypoint* ${𝐥}_{k-1}$ *generated by Algorithm 2, for which the direct segment* $\left[{𝐥}_{k-1},𝐩\right]$ *is not collision-free, there exists at least one candidate* ${𝐟}_{i}\in ℱ$ *satisfying*


$$\begin{array}{c} D_{i,k}<\infty \end{array}$$

*and the strict progress condition*


$$\begin{array}{c} \|{𝐟}_{i}-𝐩\|<\|{𝐥}_{k-1}-𝐩\|. \end{array}$$

*Then Algorithm 2 terminates after a finite number of waypoint selections and generates a finite collision-free waypoint sequence*


$$\begin{array}{c} 𝒲=\{{𝐥}_{0},{𝐥}_{1},...,{𝐥}_{k},𝐩\}. \end{array}$$

*Moreover, under the kinematic model (1) and control law (11), each active waypoint is asymptotically stable. Therefore, after the finite sequence of waypoints has been traversed, the robot asymptotically converges to the target:*


$$\begin{array}{c} \lim_{t\to \infty }𝐱(t)=𝐩. \end{array}$$

*Hence, the point-mass robot asymptotically converges to the target position.*

**Proof.** Consider an active waypoint ${𝐥}_{k}$ for which i(t)=k. While ${𝐥}_{k}$ is active, ${𝐥}_{k}$ is constant. Define the Lyapunov function


$$\begin{array}{c} V_{k}(𝐱)=\frac{1}{2}\|𝐱-{𝐥}_{k}{\|}^{2}. \end{array}$$

Differentiating $V_{k}$ along the trajectories of (1) gives


$$\begin{array}{c} {\dot{V}}_{k}=(𝐱-{𝐥}_{k}{)}^{⊤}\dot{𝐱}. \end{array}$$

Substituting the control law (11) yields


$$\begin{array}{c} {\dot{V}}_{k}=-\alpha \|𝐱-{𝐥}_{k}{\|}^{2}, \end{array}$$

where α>0. Hence,


$$\begin{array}{c} {\dot{V}}_{k}\leq 0, \end{array}$$

with equality if and only if $𝐱={𝐥}_{k}$. Therefore, each waypoint ${𝐥}_{k}$ is globally asymptotically stable while it is active.

By Proposition 1, the finite candidate set ℱ together with the strict progress condition guarantees that Algorithm 2 terminates after a finite number of waypoint selections. Under the candidate-set feasibility condition in Theorem 1, the resulting waypoint sequence


$$\begin{array}{c} 𝒲=\{{𝐥}_{0},{𝐥}_{1},...,{𝐥}_{k},𝐩\} \end{array}$$

is feasible and finite.

The switching rule activates waypoint ${𝐥}_{k+1}$ only after the robot enters the prescribed neighborhood of the current waypoint ${𝐥}_{k}$. Since ${𝐥}_{k}$ is asymptotically stable, this neighborhood is reached in finite time. Consequently, the robot progresses through the finite sequence of waypoints in succession.

In particular, after the final waypoint ${𝐥}_{k}$, the target 𝐩 becomes the active waypoint. For the final stage,


$$\begin{array}{c} {\dot{V}}_{k+1}=-\alpha \|𝐱-𝐩{\|}^{2},\;V_{k+1}=\frac{1}{2}\|𝐱-𝐩{\|}^{2}. \end{array}$$

Therefore, 𝐩 is globally asymptotically stable for the final closed-loop system, and


$$\begin{array}{c} \lim_{t\to \infty }𝐱(t)=𝐩. \end{array}$$

Hence, the point-mass robot asymptotically converges to the target position.

### Computational complexity analysis

The computational complexity of the proposed Adaptive Waypoint Generation scheme can be estimated from Algorithm 2.

During each waypoint-generation stage, the algorithm evaluates *n* candidate points. For every candidate point ${𝐟}_{i}$, collision checking is performed against all *q* obstacles for the two line segments $\left[{𝐥}_{k-1},{𝐟}_{i}\right]$ and $\left[{𝐟}_{i},{𝐟}_{i}+\gamma (𝐩-{𝐟}_{i})\right]$. Since each collision test requires checking all obstacles and the distance computation is performed in constant time, the computational cost associated with a single candidate evaluation is *O*(*q*).

Consequently, evaluating all *n* candidate points during one waypoint-generation stage requires


$$\begin{array}{c} O(nq) \end{array}$$

operations. The remaining computations, including the determination of the obstacle set ${ℐ}_{k}$, computation of the centroid ${\bar{o}}_{k}$, and evaluation of ${\hat{x}}_{k}$, require at most *O*(*q*) operations and therefore do not affect the overall complexity.

Let N−1 denote the total number of intermediate waypoints generated before the target is reached. Since the waypoint-generation procedure is repeated N−1 times, the overall computational complexity becomes


$$\begin{array}{c} O(Nnq). \end{array}$$

The memory requirement is dominated by the storage of the *n* candidate points and the *q* obstacle parameters. Therefore, the memory complexity is


$$\begin{array}{c} O(n+q). \end{array}$$

Hence, both the computational and memory requirements scale linearly with the number of candidate points, obstacles, and generated waypoints. This linear scaling makes the proposed method computationally efficient for cluttered environments.

### Parameter sensitivity analysis

To investigate the influence of the key parameters of the proposed waypoint-generation method, a parameter sensitivity analysis was conducted by independently varying the lookahead parameter γ, grid resolution $r_{g}$, and safety margin ϵ. A representative obstacle environment shown in Fig 10 was used for this analysis, with the initial position and target fixed at


$$\begin{array}{c} {𝐱}_{0}=(5,5),\;𝐩=(25,25). \end{array}$$

**Fig 10:**
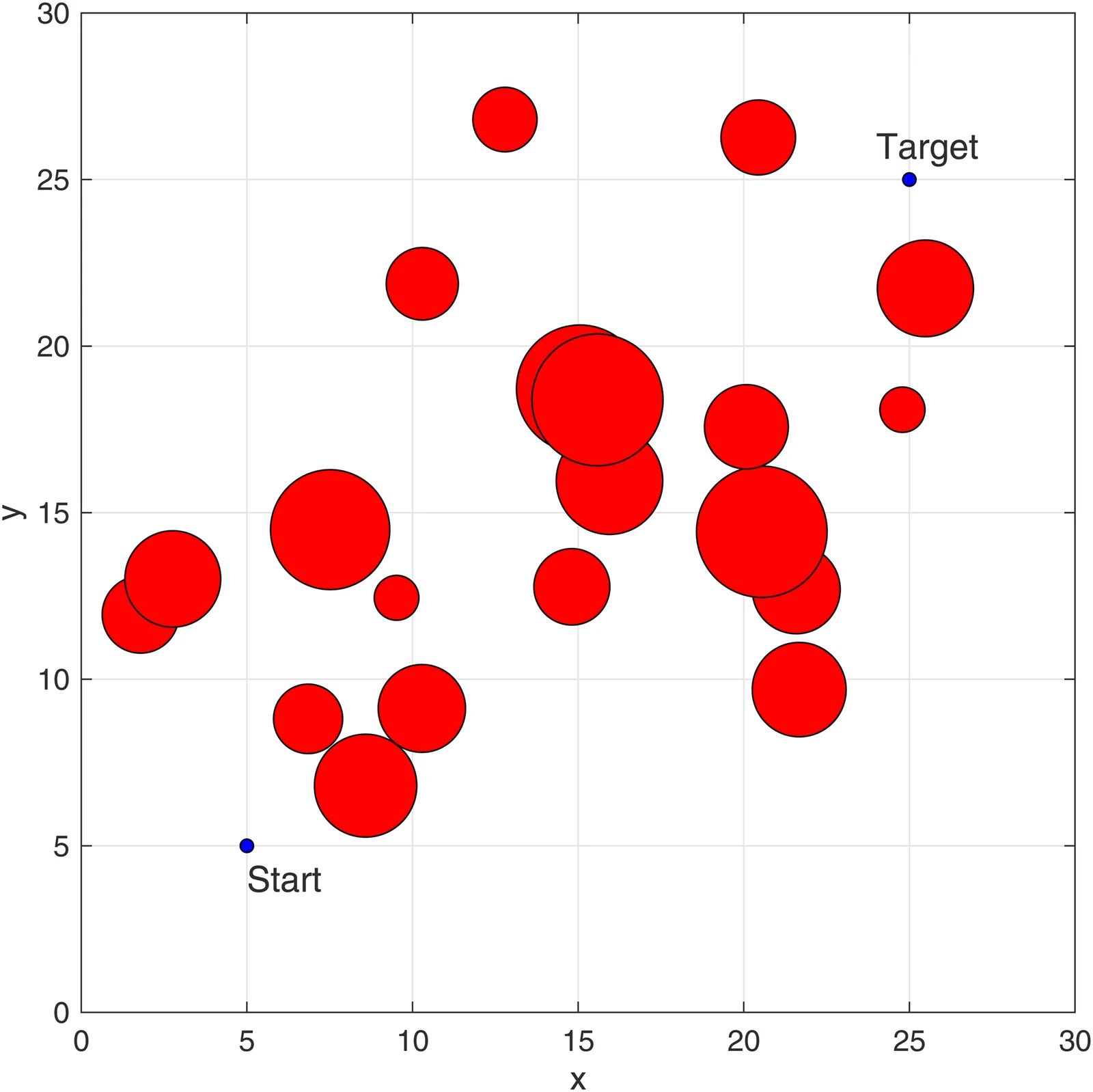
The test environment.

The obstacle configuration, robot radius $r_{P}$, and all other algorithmic parameters were kept unchanged while one parameter was varied at a time.

The sensitivity to the lookahead parameter was evaluated for


$$\begin{array}{c} \gamma \in \{0.1,\;0.25,\;0.5,\;0.75,\;1.0\}, \end{array}$$

while $r_{g}=0.5$ and ϵ=0.5 were kept fixed. As shown in Table 3, all tested values of γ produced feasible paths. However, variations in γ affected the resulting waypoint sequence and path length. The number of generated waypoints varied from one to five, while the path length varied from 34.2 to 43.4. The total time to target also varied substantially, from 194.3 to 529.3. In particular, γ=0.75 and γ=1.0 resulted in the same single-waypoint solution, with a path length of 34.2. These results indicate that although the tested values of γ maintained feasibility in this environment, the extent of the lookahead can influence waypoint selection, path efficiency, and computational cost.

**Table 3:** Parameter sensitivity analysis for the proposed waypoint-generation method.

Parameter	Value	Result	Waypoints	Path Length	Time to target
γ	0.10	Feasible	4	34.5	437.4
($r_{g}=0.5,\;\epsilon =0.5$)	0.25	Feasible	5	43.4	529.3
	0.50	Feasible	3	38.2	361.4
	0.75	Feasible	1	34.2	194.3
	1.00	Feasible	1	34.2	194.3
$r_{g}$	0.10	Feasible	3	36.4	358.1
(γ=0.5, ϵ=0.5)	0.50	Feasible	3	38.2	361.4
	1.00	Feasible	3	37.3	359.7
	1.50	Feasible	3	39.1	362.5
	2.00	Feasible	3	39.9	362.4
ϵ	0.2	Feasible	2	34.8	278.9
($\gamma =0.5,\;r_{g}=0.5$)	0.5	Feasible	3	38.2	361.4
	0.75	Feasible	3	41.5	364.9
	1	Feasible	3	43.0	364.3
	1.5	Infeasible	N/A	N/A	N/A

The effect of grid resolution was evaluated using


$$\begin{array}{c} r_{g}\in \{0.1,\;0.5,\;1.0,\;1.5,\;2.0\}, \end{array}$$

with γ=0.5 and ϵ=0.5 fixed. For all tested grid resolutions, the planner successfully generated a feasible path with three waypoints. The path length varied from 36.4 to 39.9, while the total time to target remained within the range of 358.1–362.5. These results indicate that the proposed method maintains feasibility over the tested range of grid resolutions, while the spatial discretization has a relatively limited effect on the total travel time to target for the considered environment.

Finally, the effect of the safety margin was evaluated using


$$\begin{array}{c} \epsilon \in \{0.2,\;0.5,\;0.75,\;1.0,\;1.5\}, \end{array}$$

with γ=0.5 and $r_{g}=0.5$ fixed. Increasing the safety margin resulted in a progressive increase in path length from 34.8 to 43.0 for the feasible cases. More importantly, the planner became infeasible at ϵ=1.5. This behavior is consistent with the collision condition in the proposed method, since increasing ϵ increases the required clearance around each obstacle and consequently reduces the set of feasible candidate points. Thus, the safety margin has a direct effect on the feasibility of the generated path.

Overall, the sensitivity analysis demonstrates that the proposed method remains feasible over the tested ranges of γ and grid resolution for the considered environment, while the safety margin has a more direct effect on path feasibility. The results also show that γ can influence waypoint selection and computational cost, whereas changes in grid resolution produced comparatively small variations for this particular environment. Based on these observations, γ=0.5, $r_{g}=0.5$, and ϵ=0.5 were retained as the baseline parameter values for the main simulation experiments.

### Comparative evaluation against metaheuristic waypoint planners

The trajectory examples presented in the Simulation 2 illustrate the behaviour of the proposed Adaptive Waypoint Generation (AWG) scheme in selected obstacle configurations. To provide a broader quantitative assessment, a controlled point-mass benchmark was conducted against two stochastic waypoint-planning comparators: an ant-colony-optimization-based planner (ACO) and the Stepping-Ahead Firefly Algorithm (SAFA). The purpose of this experiment was to evaluate whether the behaviour observed in individual examples remained consistent over a substantially larger set of randomly generated environments.

For each obstacle density, 100 independently generated workspaces were used, giving 400 distinct workspaces in total and 1200 method runs. Each workspace was generated once and then supplied unchanged to AWG, ACO, and SAFA. Thus, the three methods encountered identical obstacle locations and radii for a given run. Workspace and method-specific random seeds were recorded to permit exact reproduction of the experiment. Random workspaces were accepted only when a grid-based free-space connectivity check indicated that the start and target belonged to the same connected free-space region under the common inflated-obstacle model. This prevented trivially disconnected environments from being interpreted as planner failures.

All three methods shared the same point-mass model, start and target locations, collision checking, safety margin, waypoint-tracking controller, fourth-order Runge–Kutta integration, stopping tolerances, and metric definitions. Planner-specific search mechanisms and parameters were retained as part of each algorithm. The principal common settings are summarized in Table 4. Path length is reported in workspace units. Time to target is the simulated navigation time obtained from the executed trajectory using the common integration step. A run was classified as successful only if the robot reached the target tolerance without a detected collision. Mean path length and mean time to target, together with their sample standard deviations, were calculated over successful runs only.

**Table 4:** Shared settings used in the 100-run comparative point-mass benchmark.

Parameter	Setting
Workspace	[0,30]×[0,30]
Start position	(5,5)
Target position	(25,25)
Obstacle counts	10, 20, 30, and 50
Runs per obstacle count	100
Circular-obstacle radius range	[0.5,2.0]
Point-mass robot radius	0
Safety margin	0.5
Tracking control gain, α	0.1
RK4 integration step, *h*	0.1
Waypoint tolerance	0.005
Goal tolerance	0.005
Connectivity-check grid spacing	0.5
AWG candidate-grid spacing	0.5
AWG look-ahead parameter, γ	0.5
ACO/SAFA local proposal radius	3.0
ACO/SAFA evaluation budget	1000

#### Success rate, path length, and time to target.

Tables 5–8 report the requested comparison at each obstacle density. Success rate is calculated over all 100 workspaces. The path-length and time-to-target statistics are calculated only from successful runs.

**Table 5:** Comparison for environments containing 10 obstacles.

Metric	SAFA	ACO	Proposed AWG
Success rate	100%	95%	100%
Average path length	28.97	28.81	28.74
SD of path length	0.98	0.59	0.91
Average time to target (s)	329.99	323.99	143.93
SD of time to target (s)	203.57	198.70	39.90

**Table 6:** Comparison for environments containing 20 obstacles.

Metric	SAFA	ACO	Proposed AWG
Success rate	83%	77%	99%
Average path length	29.80	29.31	30.52
SD of path length	2.42	0.99	2.97
Average time to target (s)	467.18	435.57	177.27
SD of time to target (s)	284.82	176.54	47.69

**Table 7:** Comparison for environments containing 30 obstacles.

Metric	SAFA	ACO	Proposed AWG
Success rate	71%	57%	84%
Average path length	31.68	30.02	33.67
SD of path length	5.26	1.46	5.60
Average time to target (s)	667.40	512.55	222.09
SD of time to target (s)	533.97	127.92	60.60

**Table 8:** Comparison for environments containing 50 obstacles.

Metric	SAFA	ACO	Proposed AWG
Success rate	36%	19%	21%
Average path length	33.85	29.89	32.77
SD of path length	8.67	1.46	4.36
Average time to target (s)	880.82	527.01	224.99
SD of time to target (s)	794.82	120.25	59.31

At 10 obstacles, AWG and SAFA both achieved 100% success, while ACO achieved 95%. AWG also produced the lowest mean path length and the lowest mean time to target. At 20 obstacles, AWG achieved 99% success compared with 83% for SAFA and 77% for ACO. Its successful paths were slightly longer on average than those of the two stochastic planners, but its mean time to target was substantially lower. The same reliability–time trade-off was more pronounced at 30 obstacles: AWG succeeded in 84% of the workspaces, compared with 71% for SAFA and 57% for ACO, while its mean time to target remained markedly lower.

The most densely cluttered 50-obstacle case exposes an important limitation of AWG. SAFA achieved the highest success rate at 36%, while AWG and ACO achieved 21% and 19%, respectively. AWG nevertheless retained the lowest mean time to target among its successful runs. The decline in AWG success at this density was dominated by cases in which no feasible candidate waypoint could be found, rather than collision events. Across the complete 1200-run benchmark, no collision event was recorded by the shared execution and collision-checking layer. These results indicate that AWG is particularly effective in low-to-moderately cluttered environments but should not be interpreted as uniformly superior in very dense obstacle fields.

#### Matched-workspace common-success analysis.

A comparison based only on each method’s successful runs can be affected by survivor bias, because an algorithm with a lower success rate may contribute path-length and time values only from easier workspaces. To reduce this effect, a second analysis was performed using only the workspaces in which all three methods succeeded. Table 9 reports the resulting matched-workspace means.

**Table 9:** Common-success comparison using only workspaces completed successfully by AWG, ACO, and SAFA.

Obstacles	Common runs	Mean path length			Mean time to target (s)		
		AWG	ACO	SAFA	AWG	ACO	SAFA
10	95	28.67	28.81	28.82	143.14	323.99	318.58
20	76	30.04	29.31	29.37	175.26	433.64	434.60
30	49	31.65	29.90	29.83	206.34	503.50	488.55
50	8	28.73	28.91	28.91	178.85	467.03	489.09

The common-success analysis confirms that AWG’s strongest advantage is not universally shorter geometric paths, but substantially lower navigation time under the shared tracking model. On matched successful workspaces, AWG reduced mean time to target by approximately 55–64% relative to the stochastic comparators across the four obstacle densities. Path-length differences were comparatively modest and changed direction across obstacle densities. The 50-obstacle common-success subset contains only eight workspaces and should therefore be interpreted cautiously.

#### Representative matched trajectories.

Fig 11 provides representative trajectories from matched workspaces. Within each row, AWG, ACO, and SAFA were executed on the same obstacle configuration. These examples are included only to illustrate the qualitative waypoint behaviour; the quantitative conclusions are based on the full 100-run results reported above.

**Fig 11:**
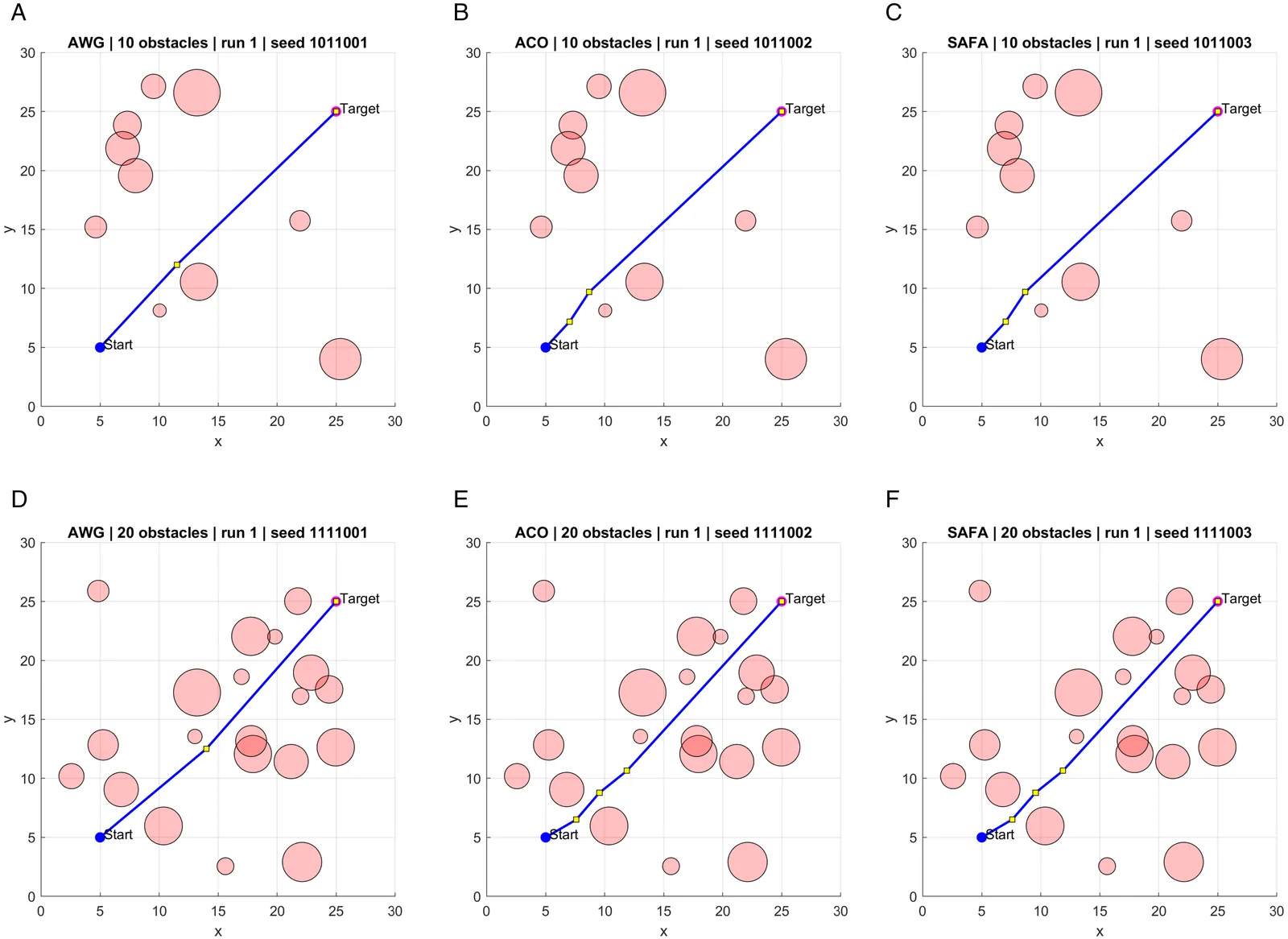
Representative matched-workspace trajectories for the 10- and 20-obstacle bench marks. Each row shows the three planners operating on the same workspace con guration and start/target pair. Yellow squares denote generated waypoints.

#### Implications of the comparative benchmark.

The expanded benchmark provides a more qualified assessment than can be obtained from individual trajectory examples alone. AWG demonstrated the highest success rate at 20 and 30 obstacles, tied with SAFA at 10 obstacles, and consistently achieved the lowest mean time to target. However, its successful paths were not consistently the shortest, and its success rate decreased sharply in the 50-obstacle case, where SAFA was more reliable. Accordingly, the principal empirical strength of AWG in the tested point-mass setting is the combination of high success in low-to-moderate clutter and comparatively rapid target attainment, rather than universal superiority in path length or dense-environment success.

The results also identify a clear boundary of the present approach. The candidate-selection mechanism can fail when the finite candidate set contains no point satisfying the forward-progress and segment-clearance conditions. This behaviour becomes more frequent as obstacle density increases. The explicit failure branch used in the benchmark records such cases rather than treating a partial trajectory as a successful run. Future work should therefore investigate adaptive candidate-grid refinement, alternative reference-point construction, and recovery strategies for cases in which the feasible candidate set is empty.

## Application: Navigating a wheel-chair robot via waypoints

Consider a planar wheelchair robot with two rear drive wheels and two front castor wheels, as shown in Fig 12.

**Fig 12:**
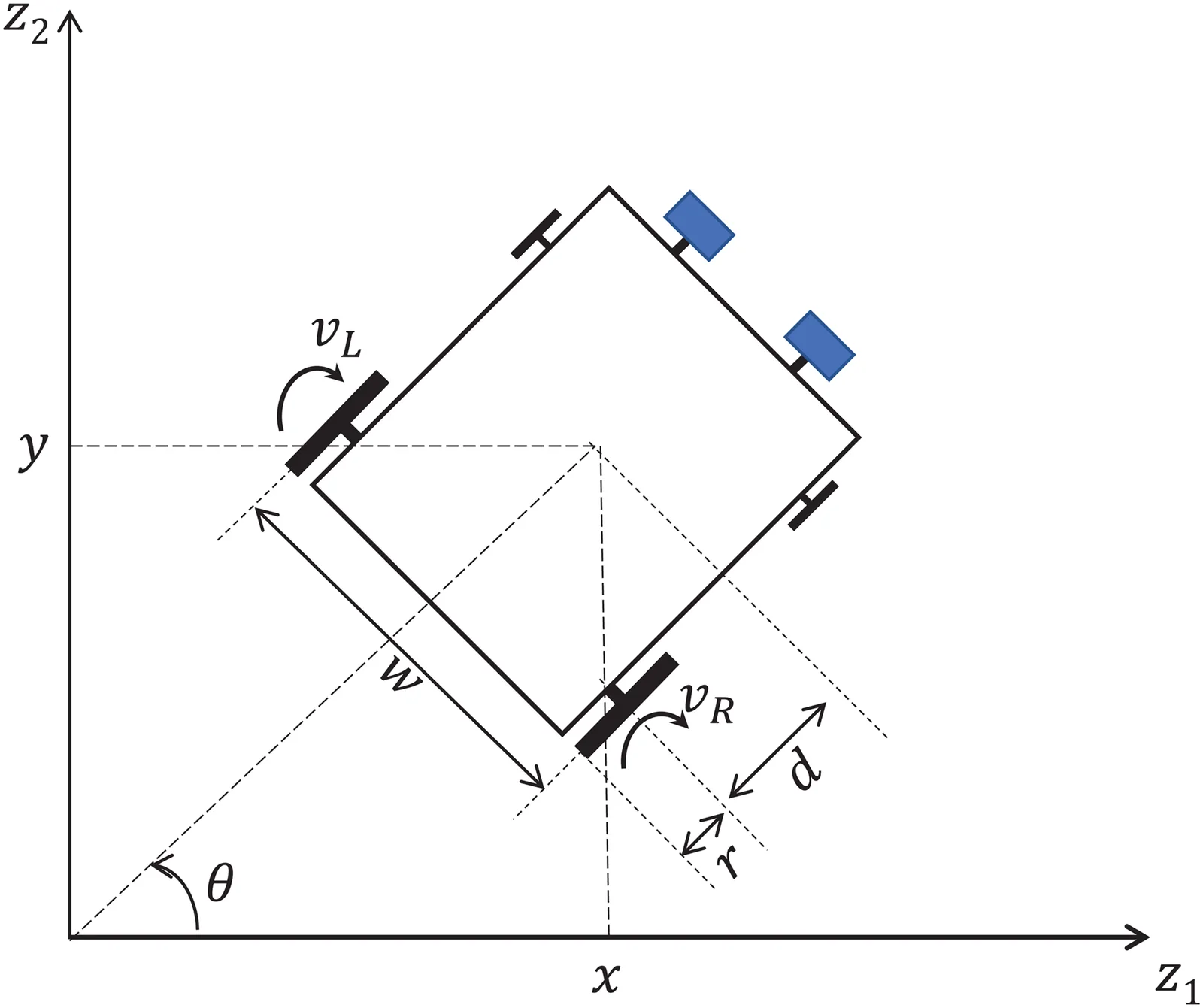
Schematic representation of a wheelchair robot.

Let (*x*,*y*) denote the Cartesian coordinates of the center of mass of the robot, *r* the radius of the rear wheels, *d* the distance from the center of mass to the line joining the rear wheels, θ the orientation of the robot relative to the *z*_1_-axis, and *w* the width of the wheelchair. The wheelchair is modelled as a nonholonomic rear-wheel-drive system with two front castor wheels. Assuming pure rolling and no lateral slip, its motion satisfies the nonholonomic constraints


$$\begin{array}{c} \dot{y}\cos\theta -\dot{x}\sin\theta -d\dot{\theta }=0, \end{array}$$

$$\begin{array}{c} \dot{x}\cos\theta +\dot{y}\sin\theta +\frac{w}{2}\dot{\theta }-rv_{R}=0, \end{array}$$

$$\begin{array}{c} \dot{x}\cos\theta +\dot{y}\sin\theta -\frac{w}{2}\dot{\theta }-rv_{L}=0, \end{array}$$

where $v_{R},v_{L}$ are the right- and left-wheel angular velocities. These constraints lead to the wheelchair kinematic model used in the present study. This formulation follows [30], where the model and Lyapunov-based stabilizing controllers were derived and analysed. Thus, the kinematic model of the wheel chair is given by [30,31].


$$\begin{array}{c} \begin{array}{cc} \dot{x} & =\frac{r}{2}(v_{R}+v_{L})\cos\theta +\frac{rd}{w}(v_{L}-v_{R}sin\theta \;,\\ \dot{y} & =\frac{r}{2}(v_{R}+v_{L})\sin\theta +\frac{rd}{w}(v_{R}-v_{L})\cos\theta \;,\\ \dot{\theta } & =\frac{r}{w}(v_{R}-v_{L}), \end{array}\} \end{array}$$ (19)

where $v_{R}$ and $v_{L}$ are the the angular velocities of the rear right and left wheels, respectively, serving as the control inputs.

To move the robot from an initial position ${𝐱}_{0}$ to a target 𝐩 via waypoints ${𝐥}_{k}=(lx_{k},ly_{k})$, we derive the control inputs using the Lyapunov-based control scheme:


$$\begin{array}{c} \begin{array}{cc} v_{R}= & \alpha \left[\left(lx_{i(t)}-x)(\frac{r}{2}\cos\theta -\frac{rd}{w}\sin\theta \right)+\left(ly_{i(t)}-y\right)\left(\frac{r}{2}\sin\theta +\frac{rd}{w}\cos\theta \right)\right],\\ v_{L}= & \beta \left[\left(lx_{i(t)}-x\right)\left(\frac{r}{2}\cos\theta +\frac{rd}{w}\sin\theta \right)+\left(ly_{i(t)}-y\right)\left(\frac{r}{2}\sin\theta -\frac{rd}{w}\cos\theta \right)\right], \end{array}\} \end{array}$$ (20)

where α>0 and β>0 are the *convergence parameters*, and


$$\begin{array}{c} i(t)=\left\{k\in \{1,...,N\}\;|\;\left\|{𝐥}_{k-1}-𝐱(0)\right\|\leq \left\|𝐱(t)-𝐱(0)\right\|<\left\|{𝐥}_{k}-𝐱(0)\right\|\right\} \end{array}$$

denotes the active waypoint index. The waypoints ${𝐥}_{k}$ are generated using Algorithm 2 with $r_{P}$ replaced by the effective robot radius


$$\begin{array}{c} r_{eff}=\sqrt{{\left(\frac{w}{2}\right)}^{2}+(r+d{)}^{2}}. \end{array}$$

For the current waypoint ${𝐥}_{k}=(lx_{k},ly_{k})$, the Cartesian tracking errors are defined as


$$\begin{array}{c} e_{x}=lx_{k}-x,\;e_{y}=ly_{k}-y. \end{array}$$

The desired translational velocities are selected as


$$\begin{array}{c} \dot{x}=\alpha e_{x},\;\dot{y}=\beta e_{y},\;\alpha ,\beta >0. \end{array}$$

Substitution into the nonholonomic kinematic model gives the corresponding wheel velocities $v_{R}$ and $v_{L}$. Using


$$\begin{array}{c} V_{i}=\frac{1}{2}(e_{x}^{2}+e_{y}^{2}), \end{array}$$

gives


$$\begin{array}{c} {\dot{V}}_{i}=-\alpha e_{x}^{2}-\beta e_{y}^{2}\leq 0, \end{array}$$

which verifies convergence to the active waypoint under the stated assumptions.

### Simulation 3

The effectiveness of the proposed waypoint-based navigation strategy is evaluated using a planar wheelchair robot operating in an obstacle-cluttered workspace. Four simulation scenarios are considered, each involving navigation from an initial configuration to a target position. The simulation parameters are summarized as follows:

- Robot dimensions: *r* = 0.3, *d* = 0.25, and *w* = 0.65 meters.
- Initial positions and orientations, and target position are given in Table 10:

**Table 10:** Simulation scenarios and corresponding initial conditions.

Scenario	Initial Position	Initial Orientation	Target Position
	$\left(x_{0},y_{0}\right)$	θ(0) (rad)	$\left(p_{1},p_{2}\right)$
1	(1,1)	0	(9,9)
2	(9,1)	π/2	(1,9)
3	(1,9)	0	(9,1)
4	(9,9)	π	(1,1)

- Convergence parameters: α=β=0.2.
- Workspace boundaries: $0\leq z_{1}\leq 10$ and $0\leq z_{2}\leq 10$.

Figs 13–16 illustrate the navigation trajectories of the wheelchair robot generated by the proposed Adaptive Waypoint Generation (AWG) method and the conventional Artificial Potential Field (APF) approach in environments of increasing complexity. The blue trajectories correspond to the proposed method, while the brown trajectories represent the APF method. In all scenarios, the proposed method successfully guides the wheelchair from the start position to the target while maintaining safe clearance from obstacles.

**Fig 13:**
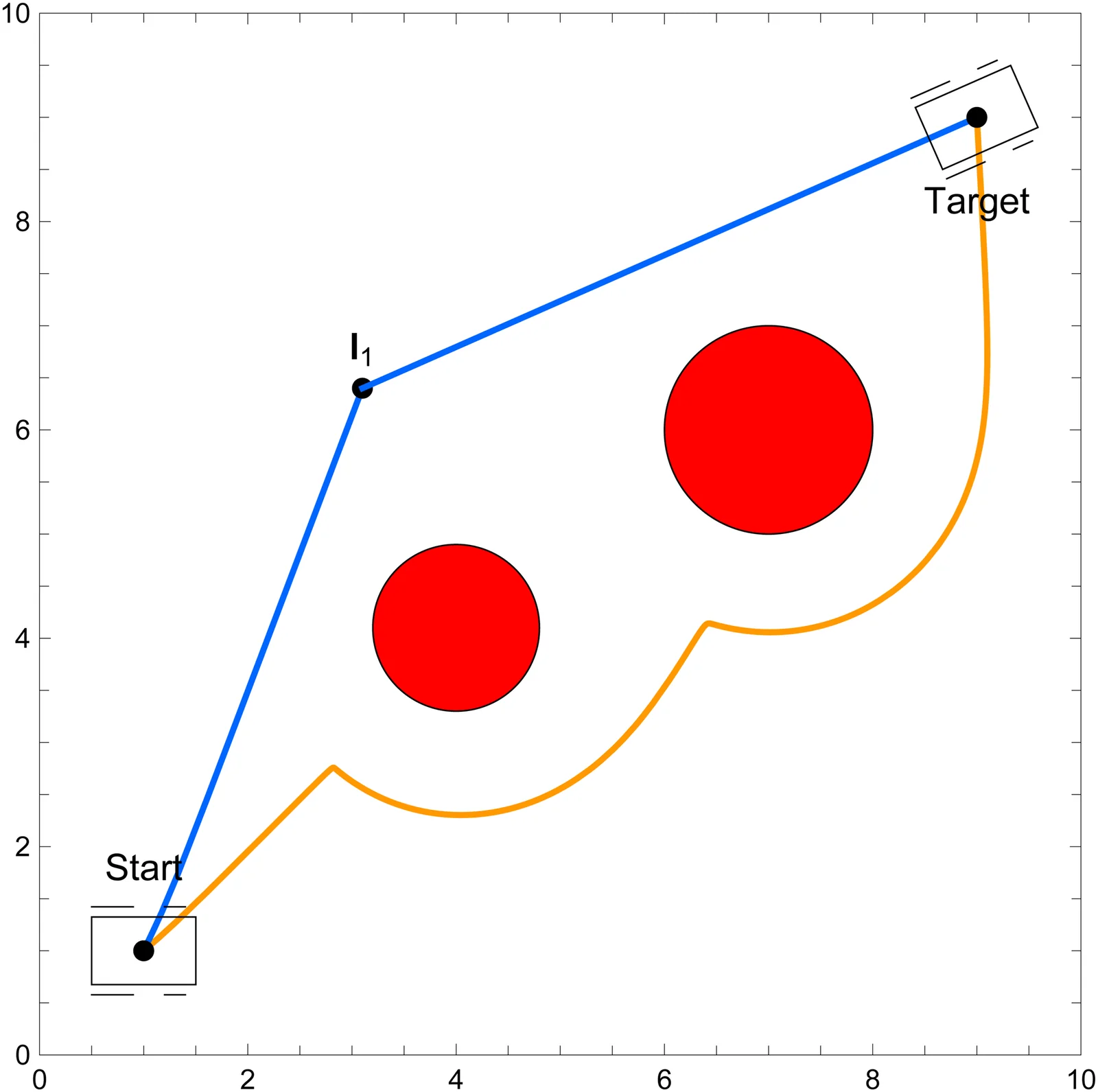
Scenario 1: Navigation trajectories in a workspace containing 2 obstacles of random sizes and positions. Blue – proposed method, brown – APF method.

**Fig 14:**
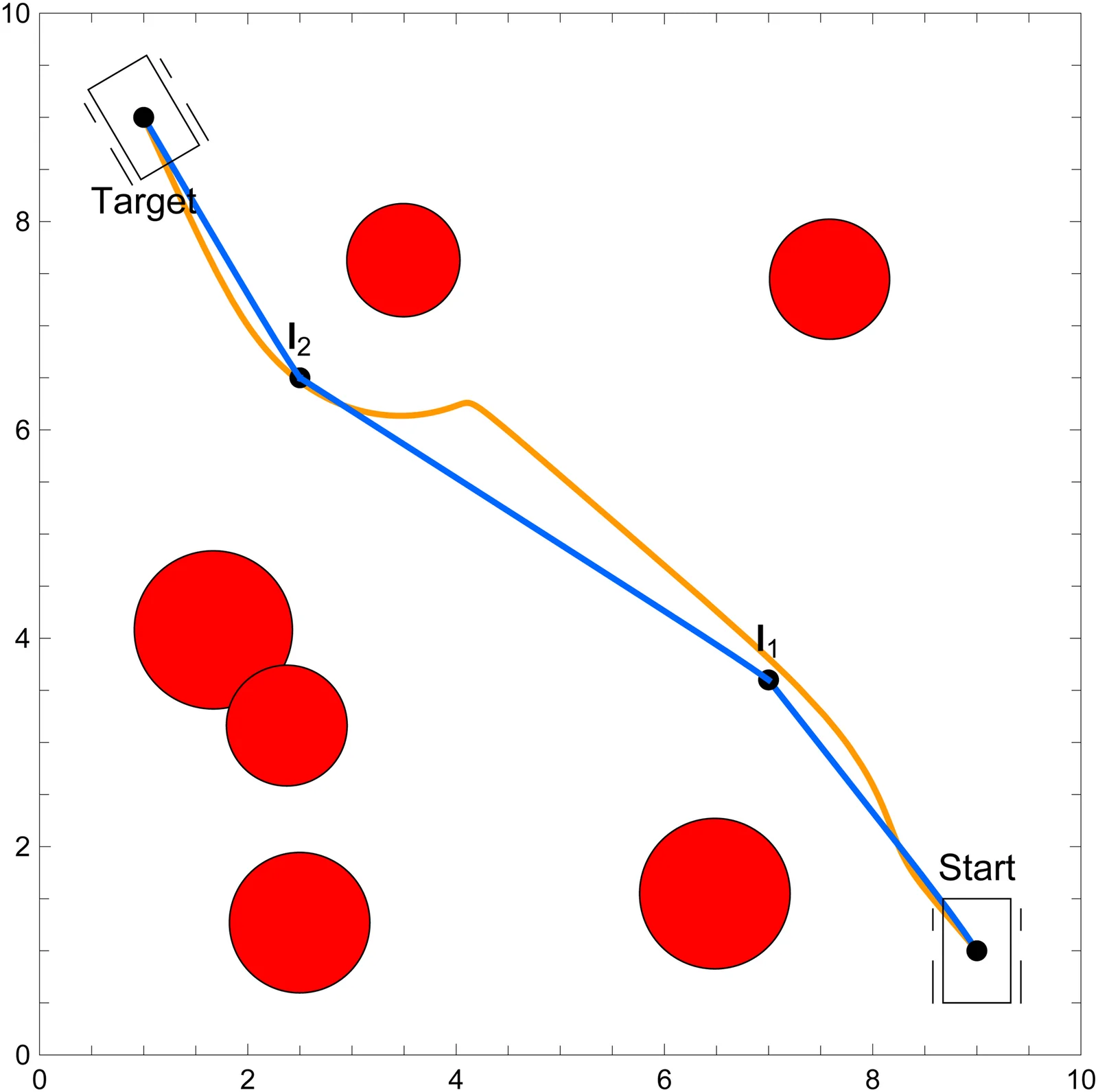
Scenario 2: Navigation trajectories in a workspace containing 6 obstacles of random sizes and positions. Blue – proposed method, brown – APF method.

**Fig 15:**
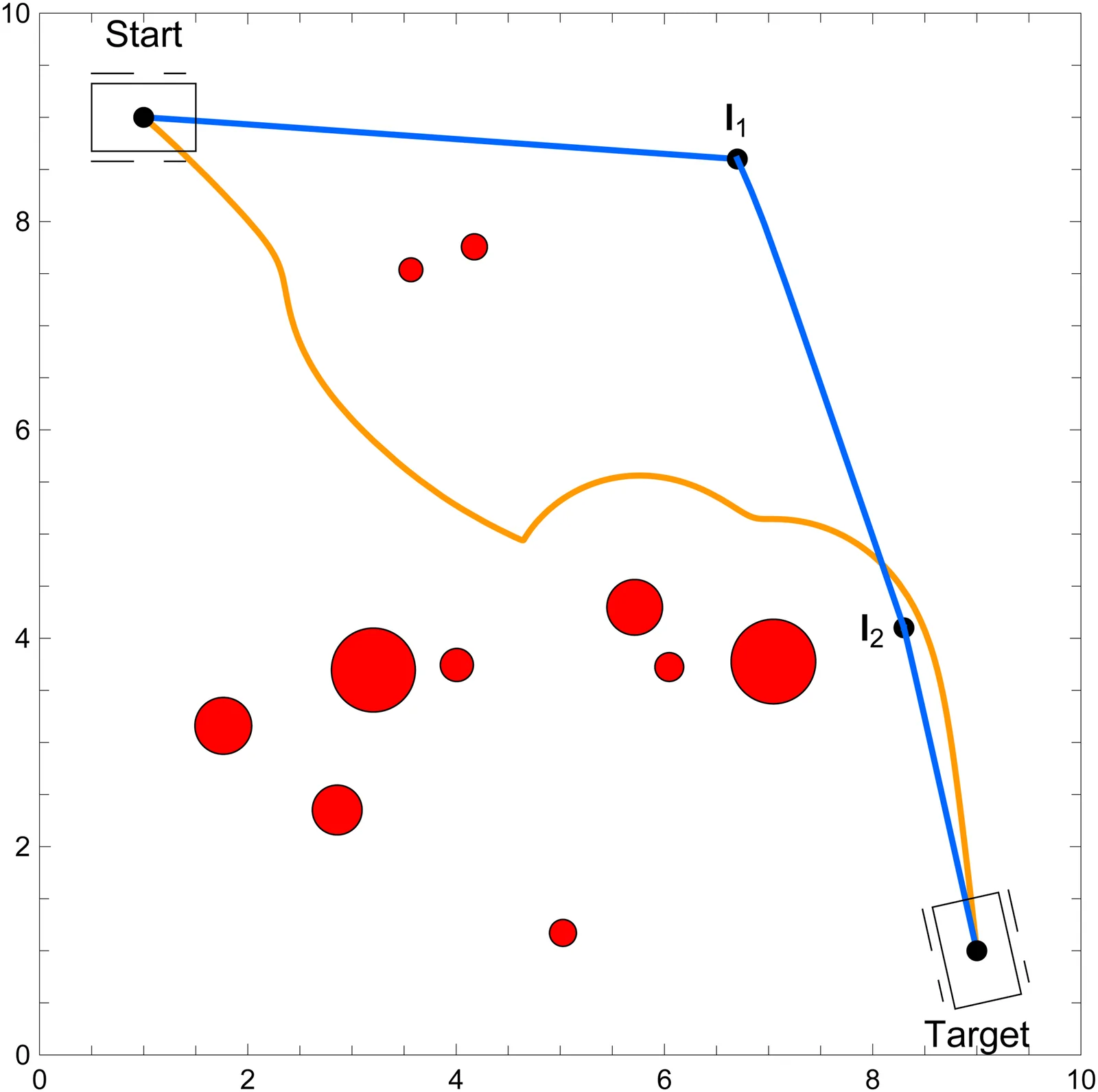
Scenario 1: Navigation trajectories in a workspace containing 10 obstacles of random sizes and positions. Blue – proposed method, brown – APF method.

**Fig 16:**
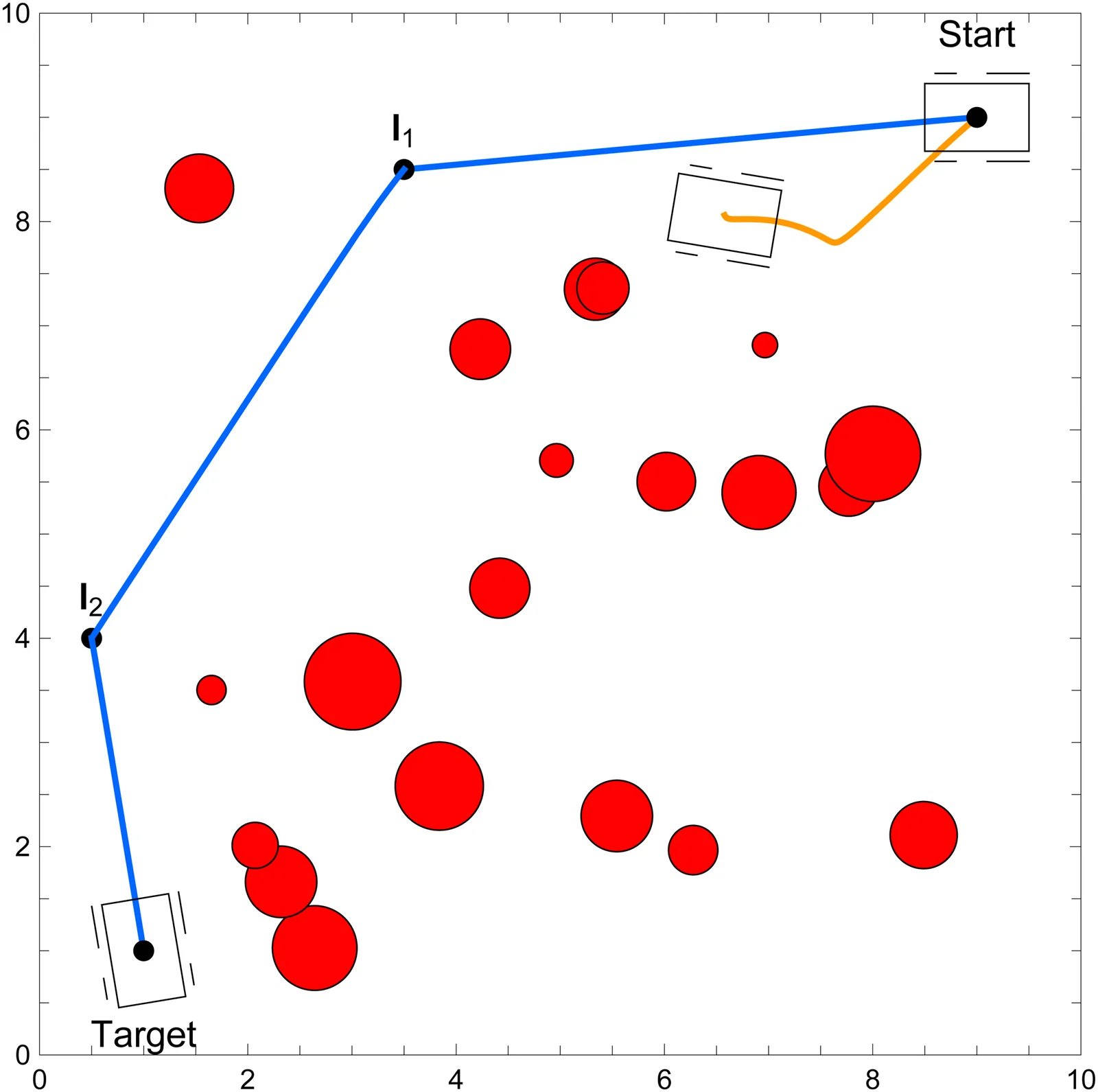
Scenario 2: Navigation trajectories in a workspace containing 20 obstacles of random sizes and positions. Blue – proposed method, brown – APF method.

In Fig 13, the environment contains only two obstacles. Both methods successfully reach the target position. The APF method generates a smooth trajectory that closely follows the obstacle boundaries. In contrast, the proposed method navigates through an intermediate waypoint (${𝐥}_{1}$), producing a more direct trajectory toward the goal. Both methods enable the wheelchair to reache the destination efficiently while maintaining a safe distance from obstacles.

Fig 14 presents a more cluttered environment containing six obstacles. The proposed method successfully computes intermediate waypoints (${𝐥}_{1}$ and ${𝐥}_{2}$) that guide the wheelchair through obstacle-free regions toward the target. The generated trajectory remains smooth and collision-free. The APF trajectory exhibits stronger deviations caused by the cumulative repulsive effects of nearby obstacles, resulting in a less direct route. Although APF still reaches the target in this scenario, its path shows increased curvature and longer traversal time. The results indicate that the waypoint-based strategy improves navigation efficiency in moderately complex environments.

As the obstacle density increases to ten obstacles in Fig 15, the advantage of the proposed method becomes more evident. The wheelchair follows a structured path through two intermediate waypoints, allowing it to avoid congested regions and proceed toward the goal in a predictable manner. The APF trajectory experiences significant turns and detours due to the repulsive forces from multiple obstacles. These effects increase path complexity and may reduce navigation efficiency. The proposed method demonstrates greater robustness by maintaining a clear and stable trajectory despite the increased environmental complexity.

Fig 16 depicts a highly cluttered workspace containing twenty randomly distributed obstacles. The proposed method continues to generate a feasible trajectory by strategically selecting intermediate waypoints that steer the wheelchair through available free space. The resulting path remains collision-free and successfully reaches the target despite the dense obstacle distribution. In contrast, the APF method struggles to progress toward the goal and becomes trapped in a local minimum near the starting region. Consequently, APF fails to generate a complete path to the target. This result highlights one of the principal limitations of conventional APF navigation and demonstrates the effectiveness of the proposed Adaptive Waypoint Generation strategy in overcoming local minima problems.

The quantitative results presented in Table 11 support these observations. In Scenarios 1 and 2, the proposed method achieved both shorter path lengths and lower navigation times than APF. Although APF generated a shorter path in Scenario 3, it required substantially more time to reach the target. In Scenario 4, the proposed method successfully completed the navigation task, whereas APF failed to find a feasible path. Overall, the results demonstrate that the proposed method provides more reliable navigation in environments containing APF local-minimum configurations, while its travel-time performance is scenario dependent..”

**Table 11:** Performance comparison for wheelchair robot navigation.

Scenario	Method	Path Length (units)	Time Taken (units)
1	Proposed	12.240	907
	APF	12.832	2116
2	Proposed	11.547	985
	APF	11.949	1837
3	Proposed	13.664	1117
	APF	10.159	2425
4	Proposed	13.969	1124
	APF	Failed	Failed

Overall, the qualitative trajectory analysis and quantitative performance metrics confirm that the proposed Adaptive Waypoint Generation strategy effectively addresses the limitations of conventional APF navigation. By overcoming local minima while maintaining safe obstacle avoidance, the method enables robust and efficient autonomous wheelchair navigation in cluttered environments.

## Discussion

The simulation results demonstrate that the proposed waypoint-generation strategy can effectively address local-minimum situations commonly encountered with the artificial potential field (APF) method. Unlike conventional APF approaches, which rely solely on attractive and repulsive potential forces to guide a robot toward its target, the proposed method introduces automatically generated intermediate waypoints that act as temporary navigation objectives. These waypoints redirect the robot around obstacle regions while maintaining goal-directed motion.

The numerical experiments reveal two important observations. First, in obstacle-free environments, or when the straight-line path from the start position to the goal does not intersect any inflated obstacle region, intermediate waypoints are not required. Under such conditions, a feasible collision-free path already exists, allowing the attractive potential to guide the robot directly toward the goal. Consequently, both the proposed method and the conventional APF method can produce similar trajectories and comparable performance. Second, in the tested single-obstacle and multi-obstacle scenarios, the APF trajectories became trapped in local-minimum regions when the attractive and repulsive forces balanced each other. In contrast, the proposed waypoint-generation procedure identified feasible intermediate locations that redirected the robot around the problematic regions. The robot consequently reached the target in these tested cases without becoming trapped. These results indicate that the proposed approach can improve navigation reliability in the considered cluttered environments.

An additional consideration is the computational and trajectory trade-off between the proposed method and APF. The proposed framework explicitly performs collision checking and, when necessary, generates intermediate waypoints before the robot proceeds toward the target. This additional planning step can result in longer trajectories and higher travel times than those produced by APF. Indeed, the simulation results show that the proposed method generally requires more travel time than the APF baseline. This behavior is expected because APF directly generates a continuous control action from the potential field, whereas the proposed approach introduces additional waypoint transitions to obtain explicitly collision-free path segments. Thus, the proposed method is not intended to outperform APF in travel time under all conditions. Rather, the results highlight a trade-off between direct potential-field motion and explicit waypoint-based obstacle avoidance. Incorporating path length, travel time, or control effort directly into the waypoint-selection criterion is a potential direction for reducing this additional cost.

The waypoint-generation procedure also has a relatively simple geometric structure, since candidate selection is based on distance calculations and explicit collision checking. Nevertheless, the computational cost depends on the number of candidate points and the number of obstacles that must be evaluated. Increasing the size of the candidate set can provide more opportunities for finding suitable waypoints, but also increases the number of collision-checking operations. Therefore, the computational efficiency of the method should not be interpreted as independent of the candidate-set size. Adaptive candidate sampling and more efficient spatial collision checking are potential approaches for improving computational performance in larger environments.

The simulations reveal an important distinction between path length and travel time. In the point-mass experiment involving 15 obstacles, the proposed AWG method generated a slightly shorter path than APF (31.69 versus 33.57 units) but required substantially more travel time (268.9 versus 92.4 time units). This increase is primarily associated with the sequential waypoint-tracking mechanism, in which the controller converges toward each intermediate waypoint before proceeding to the next. In contrast, APF continuously directs the robot toward the final target and therefore avoids the additional convergence time associated with intermediate goals. Accordingly, the proposed method should not be interpreted as universally minimizing travel time; rather, it introduces a trade-off between direct continuous motion and reliable navigation through potentially problematic obstacle configurations. This trade-off is also scenario dependent. In the wheelchair simulations, the proposed method required less time than APF in Scenarios 1–3, while APF failed to reach the target in Scenario 4. These results indicate that the primary contribution of the proposed method is improved feasibility and robustness against local-minimum failures rather than guaranteed minimization of travel time. Although the generated trajectories were collision-free in the tested scenarios, the current method does not explicitly optimize trajectory smoothness, curvature, energy consumption, or control effort. Future work will therefore investigate multi-objective waypoint-generation and control formulations that jointly consider collision clearance, path length, travel time, trajectory smoothness, curvature, and control effort or energy consumption, with the aim of improving trajectory efficiency while maintaining reliable obstacle avoidance.

A comparison with metaheuristic waypoint planners, ACO and SAFA further highlights the performance characteristics of the proposed AWG method. AWG achieved success rates of 100%, 99%, and 84% for 10, 20, and 30 obstacles, respectively, compared with 95%, 77%, and 57% for ACO and 100%, 83%, and 71% for SAFA. However, at 50 obstacles, AWG achieved 21% success compared with 19% for ACO and 36% for SAFA, indicating that its effectiveness decreases in highly cluttered environments. Among the common-success cases, the three methods produced comparable path lengths, whereas AWG consistently required substantially less time to reach the target. The mean target-reaching time for AWG ranged from 143.14 to 206.34 s, compared with 323.99–503.50 s for ACO and 318.58–489.09 s for SAFA. This difference suggests that the explicit geometric waypoint-generation strategy can reduce the time associated with iterative metaheuristic path planning while maintaining comparable path lengths. Nevertheless, the results at 50 obstacles should be interpreted cautiously because only eight common-success cases were available. Overall, the comparison indicates that AWG provides effective navigation in low-to-moderately cluttered environments, while further improvements are required to maintain reliable performance as obstacle density increases.

The applicability of the waypoint-generation framework to different robot models was investigated by considering both a point-mass robot and a nonholonomic wheelchair robot. The successful simulations in these two cases suggest that the waypoint-generation concept can be combined with different motion-control laws. However, this observation should not be interpreted as a general guarantee of model-independent performance for arbitrary robotic platforms. In particular, dynamic constraints, actuator limitations, perception errors, and model uncertainty may affect the performance of an implementation on a physical robot. For the wheelchair, obstacle clearance is computed using the effective robot radius ($r_{eff}=\sqrt{w^{2}/4+(r+d{)}^{2}}$), which accounts for the wheelchair geometry during waypoint generation. Thus, the line segments connecting consecutive waypoints satisfy the required geometric clearance. However, because the wheelchair follows a nonholonomic trajectory, the swept footprint along the actual trajectory is not formally certified by the segment-clearance condition alone. The presented simulations verify collision-free trajectories, while a formal swept-footprint collision guarantee is left for future work.

Another important limitation of the present study is that the obstacle configuration is assumed to be static and fully known. This assumption allows the effectiveness of the proposed Adaptive Waypoint Generation (AWG) strategy to be evaluated independently of perception and state-estimation errors; however, practical mobile robots typically operate with incomplete and uncertain information, and obstacles may move during navigation. The proposed framework can nevertheless provide a basis for extension to such environments because waypoint generation is performed adaptively through repeated segment-wise collision checking. In a dynamic implementation, the obstacle set could be updated at each planning cycle using the latest sensor measurements, and the waypoint-generation procedure could be executed in a receding-horizon manner whenever the currently planned segment becomes infeasible. For moving obstacles, short-term obstacle motion predictions could be incorporated into the collision-checking process by considering their predicted occupied regions over the planning horizon. Sensor and localization uncertainty could similarly be accommodated by increasing the effective obstacle-clearance margin. In particular, the current clearance condition ($ro_{l}+r_{P}+\epsilon$) could be augmented by an uncertainty margin (δ), giving ($ro_{l}+r_{P}+\epsilon +\delta$), where (δ) represents a bound or confidence-dependent measure of sensing and localization uncertainty. Such an approach would make the waypoint-generation process more conservative in regions where obstacle estimates are uncertain. These extensions are conceptually compatible with the present framework but have not been experimentally evaluated in this study and therefore constitute an important direction for future work.

Overall, the results demonstrate that the proposed waypoint-generation strategy provides an effective mechanism for addressing local-minimum situations in the tested static obstacle environments. The principal advantage of the approach is the explicit generation and collision checking of intermediate navigation objectives, while its main trade-offs include additional travel time, dependence on the candidate-set design, and the assumption of known static obstacles. These limitations define clear directions for future work, including dynamic-obstacle handling, sensor-uncertainty modeling, adaptive candidate generation, trajectory-efficiency optimization, and hardware-based validation.

## Conclusion

This paper presents a novel approach for generating waypoints to facilitate reliable robot navigation. The proposed optimization-based algorithm identifies points in the robot’s workspace that act as subgoals, guiding the robot safely toward its target. By ensuring that these waypoints lie in obstacle-free regions, the robot can move between them without collisions. The method also effectively addresses the local minima problem, which commonly limits the performance of the Artificial Potential Field (APF) method.

The algorithm is versatile and can be applied to a wide range of robotic systems beyond point-mass models. As an illustrative example, it was successfully employed to navigate a wheelchair robot from an initial configuration to a target position via intermediate waypoints. Matlab-based simulations validated the effectiveness of the approach in both single- and multi-obstacle environments.

Future research could extend this method to three-dimensional robotic platforms, including mobile robotic arms, drones, quadrotors, and other unmanned aerial vehicles, potentially incorporating additional objectives such as path smoothness, energy efficiency, or dynamic obstacle avoidance.

## Supporting information

S1 file Mathematica and Matlab. (ZIP)
